# Kava consumption and the rise of sociopolitical complexity in Oceania

**DOI:** 10.1073/pnas.2521658123

**Published:** 2026-02-23

**Authors:** Václav Hrnčíř, Oliver Sheehan, Scott Claessens, Russell D. Gray

**Affiliations:** ^a^Department of Linguistic and Cultural Evolution, Max Planck Institute for Evolutionary Anthropology, Leipzig 04103, Germany; ^b^School of Psychology, University of Auckland, Auckland 1142, New Zealand; ^c^School of Psychology, University of Kent, Canterbury CT2 7NZ, United Kingdom

**Keywords:** kava, cross-cultural, sociopolitical complexity, cultural evolution, Oceania

## Abstract

Humans have been using psychoactive substances for millennia, despite their potential negative health and social consequences. According to some scholars, our craving for mind-altering drugs is an evolutionary mistake—a hijacking of our reward system. In contrast, the “drunk hypothesis” argues that intoxication has been adaptive and essential for the rise of large-scale societies because it promotes social bonding, increases cooperation, alleviates stress, and enhances human creativity. Here, we test this hypothesis using the example of kava, a traditional Pacific beverage with a range of psychoactive effects, made from the root of *Piper methysticum*. Our analysis of 83 Oceanic-speaking societies shows a positive relationship between traditional kava consumption and both political complexity and social stratification. However, the results are not robust to controls for nonindependence. Moreover, we found no evidence of coevolution between kava drinking and either of the two sociopolitical traits after controlling for spatial nonindependence. Despite the cultural significance of kava in many Pacific societies, our results suggest that its consumption was unlikely to have been a major driver of sociopolitical complexity, underscoring the importance of controlling for nonindependence in cross-cultural studies.

## Significance

People’s attitude toward psychoactive substances is highly ambivalent, with an ongoing debate about their possible adaptive or maladaptive role in human evolution. Here, we present a cross-cultural study testing the hypothesis that consumption of the mind-altering beverage kava facilitated the emergence of complex, hierarchical societies in Oceania. Despite finding a positive, albeit uncertain, correlation between kava drinking and sociopolitical complexity, we found no evidence for the coevolution of these cultural traits. The results suggest that kava was not the primary factor explaining the rise of sociopolitical complexity in this region. However, ethnographic evidence shows that kava played a significant role in maintaining and strengthening existing political and social relationships.

Humans around the world have been consuming psychoactive substances for millennia, including cannabis in Central Asia, betel nuts in Southeast Asia, opium poppy in the Mediterranean, tobacco in the Americas, coca leaves in the Andes, ayahuasca in Amazonia, pituri in Australia, and psychedelic mushrooms in Mesoamerica and Siberia (1, 2). On most continents, people have also consumed traditional alcoholic beverages fermented from honey, fruit, palm sap, or grain (3). It is unknown when our ancestors first began experimenting with intoxicants, or which were the earliest. However, given that many other animals, such as insects, birds, cats, and wild primates, deliberately and regularly get drunk and high in their natural environments (4, 5), the history of human intoxication may be very deep.

The widespread use of intoxicants contrasts with their many negative health and social consequences. These relate in particular to excessive use, which is fueled in modern times by relatively straightforward and unlimited availability, high concentrations of psychoactive substances, and a tendency toward solitary consumption. Over 3 million people are estimated to die each year due to alcohol and drug use (6), and many more suffer from drug-related crime, violence, domestic abuse, and poor work performance.

Many scholars have tried to explain humanity’s paradoxical relationship with intoxication. According to some, our craving for mind-altering drugs is an evolutionary mistake. Either it “hijacks” our reward systems, which were initially designed to encourage other, more adaptive behaviors (7) or their formerly adaptive consumption became maladaptive once humans began to produce drugs on a large scale (8). The adaptive attraction to alcohol, for example, may stem from the importance of ethanol-rich fruits in the diet of our hominid ancestors (9, 10), while the seeking out and consumption of neurotoxic plants, such as tobacco, cannabis, and betel nut, might be an evolved self-medication strategy to defend against pathogens, especially those that infect the central nervous system (11, 12). Other potential fitness benefits derived from the ability to utilize drugs include improved performance from stimulants, pain relief from analgesics and opiates, elevated mood from antidepressants, and enhanced social and cognitive capacities from psychedelics (13, 14).

The recently proposed “drunk hypothesis” (2) states that the use of intoxicants, especially alcohol, facilitates life in a human ecological niche—one that requires cooperation and cultural innovations—and has been essential for the rise and maintenance of large-scale complex societies. By temporarily downregulating the prefrontal cortex and activating the endorphin system, alcohol makes us more trusting, emotional, creative, and relaxed (15–17), allowing us to more easily cooperate, bond and identify with strangers, and cope with stressful life in crowded, hierarchical societies. These cognitive effects are often intensified by a combination of intoxication with various nonchemical bonding practices, such as feasting, dancing, and group singing (18–20).

The potential role of alcohol in the evolution of complex societies has been supported by a cross-cultural study (3) that found a positive, although modest, effect of traditional nondistilled fermented beverages on the rise of political complexity. However, the hypothesis does not apply only to alcoholic intoxication. In Oceania, where alcohol was unknown until the time of European contact (21), it has been proposed that kava may have played a similar role, as its consumption also strengthens social bonds and cooperation, reduces anxiety, and is a source of creative inspiration (2). On the other hand, some of its pharmacological effects are different—more calming than euphoric, and without the significant cognitive impairment and behavioral disinhibition typical of alcohol.

To test this hypothesis, we analyzed the association between traditional kava drinking and sociopolitical complexity and examined whether and how these cultural traits coevolved in Oceanic societies.

## Kava Consumption in Oceania.

The name kava refers to both the plant *Piper methysticum* and the psychoactive beverage prepared from its rootstock (22, 23). The plant is a hardy, slow-growing perennial shrub that belongs to the Piperaceae family, which also includes black pepper (*Piper nigrum*) and betel (*Piper betle*). *Piper methysticum* is not a distinct species but rather a group of sterile cultivars, selected and cloned from its wild progenitor, *Piper wichmannii*. Domesticated cultivars are incapable of sexual reproduction; they are propagated exclusively due to human effort using stem cuttings. The term kava probably derives from a Proto-Oceanic term *kawaRi that referred to “roots with special properties” (24). Other names for kava include “*ava* in Samoa,” “*awa* in Hawai”i, *yaqona* in Fiji, *maloku* in northern Vanuatu, *sakau* on Pohnpei, and *seka* on Kosrae.

Limited archaeobotanical evidence suggests that occupants of the Bismarck Archipelago may have experimented with the wild form of kava (*Piper wichmanii*) already in the early Lapita period (25). According to Brunton (26), this is also the region where *Piper methysticum* was first domesticated. However, the latest botanical, genetic, and chemical evidence places kava’s origin rather in northern Vanuatu, approximately 3,000 y ago (23, 27). From there, kava likely spread eastward to Polynesia and westward into areas of New Guinea and Micronesia, northward as far as Hawaii, and southward as far as New Zealand. In the latter case, however, its cultivation did not take off because of the cold climate. Only the name *kawa-kawa* survived, which Māori gave to another species of the family Piperaceae, *Macropiper excelsum.*

Kava is cultivated for its psychoactive ingredients, the kavalactones, which are most concentrated in the underground organs of the plant (28). Traditionally, these are consumed in the form of a drink prepared by infusing the chewed, ground, or pounded roots and stumps with cold water. The resultant drink has a broad spectrum of pharmacological properties, including anxiolytic, sedative, soporific, analgesic, anesthetic, and muscle relaxant effects (22, 23, 29). Its psychoactive potency varies from very weak to quite strong, depending on the plant variety, growing conditions, the way the drink is prepared, and the quantity consumed during a drinking session. Traditionally, kava is consumed at social gatherings, as well as during religious and cultural ceremonies, where it evokes an atmosphere of calm, relaxation, and sociability among drinkers. Other positive effects include mild euphoria, reduced anxiety, and feelings of happiness. In large doses, it may induce sleepiness, impair motor skills, and provoke photophobia, diplopia, and nausea. Heavy consumption can result in the presence of a reversible scaly skin rash known as “kava dermopathy,” weight loss, indigestion, and general poor health (30). However, kava is neither hallucinogenic nor stupefying, and unlike alcohol, it has no deleterious effects on cognition nor is it physically addictive (29, 31). Although concerns over rare cases of hepatotoxicity led to the restriction of kava in several countries, the consumption of the kava beverage is generally considered safe (30).

## Kava and Sociopolitical Complexity.

Kava plays a significant role in the religious, social, political, and economic spheres throughout Oceania, although the details of its functions vary from society to society (22, 23, 27, 32). People use kava intoxication to communicate with gods and ancestral spirits, welcome guests and socialize with friends, celebrate significant life events, treat physical and mental pains, install chiefs and validate titles, make peace with enemies, and reinforce group identity and social cohesion. Like some other psychoactive substances, kava is valued for its transformative effects and its ability to connect humans with the supernatural realm, through which new wisdom, visions, and inspiration come. It is a social drug that facilitates discussion, promotes sociability, and evokes feelings of camaraderie. The preparation and consumption of kava is always a group activity. In Fiji, drinking kava alone is even considered dangerous and suspicious, associated with witchcraft (33).

In many societies, such as the chiefdoms of Samoa, Tonga, and Fiji, the traditional kava ceremonies serve to reaffirm and reinforce the existing social hierarchies (32). Various protocols govern where people sit, how kava is prepared and served, and also the order in which it is drunk; all of which is strictly dictated by social rank and status titles. By sharing kava, drinkers accept and confirm the existing political hierarchy among titleholders, as well as general relationships of inequality between participants. An observational study of kava drinking rituals in a Fijian community revealed that participation in rituals and excessive kava consumption are crucial for both achieving prestige and status within the community and fostering social alliances. In contrast, men who refrain from participating in kava ceremonies are excluded from many social benefits, such as cooperative farming (34).

Kava is an important gift within traditional exchange systems. It serves to reduce hostility and ensure peacefulness and tranquility, both in interpersonal and intergroup relationships. Exchanges of kava lubricate the prevailing political and social order. Commoners give kava in tribute to their chiefs, while leaders use it to attract supporters (23). Islanders drink it to ratify agreements and to celebrate the formation of new political alliances. For many Oceanic peoples, kava also symbolizes a shared national ethnocultural identity (35).

**Fig. 1.**
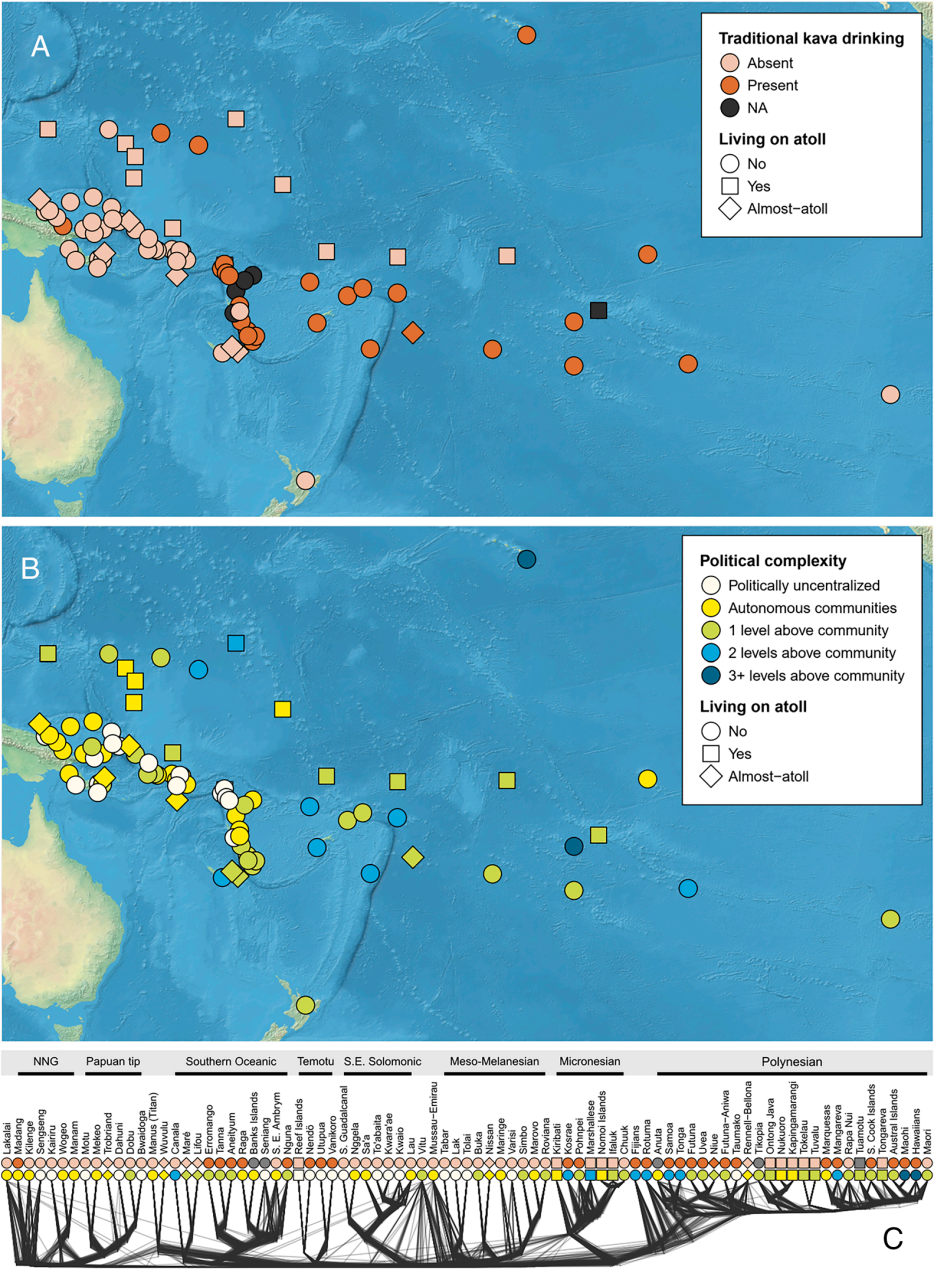
Distribution of (*A*) traditional kava drinking and (*B*) political complexity in 83 Oceanic-speaking societies. Panel (*C*) shows both cultural traits plotted on the language phylogeny based on 100 randomly drawn posterior trees from Gray et al. (39). NNG, North New Guinea linkage. (Images created using map data from Natural Earth, www.naturalearthdata.com).

While kava has played a crucial role in social and political institutions in recent history, its potential as an active driver of sociopolitical complexity in the cultural evolution of Oceanic societies has not yet been fully explored. This study attempts to fill this gap by collecting and analyzing ethnographic data from 83 Oceanic-speaking societies on the presence of traditional kava drinking and two commonly used measures of complexity (36–38)—political complexity (i.e., the number of jurisdictional levels in the society) and social stratification (i.e., the relative complexity of graded and hereditary status distinctions in the society; Fig. 1 and *SI Appendix*, Fig. S1)—as they were before the impact of European contact, i.e., missionization and colonization.

To understand the association between kava and both sociopolitical traits, we first employed causal inference methods (40) and Bayesian regression models (41). We modeled political complexity and social stratification as response variables and kava as the predictor variable, controlling for potential confounding effects of spatial proximity, common cultural ancestry, and atolls. We included the first two confounders because it is known that geographically close and historically related societies are more likely to be culturally similar (42, 43), which could lead to spurious interpretations of the results (44). Atolls are also potential confounders, because this type of island is environmentally unsuitable for kava cultivation (26), and the generally poor resources on atolls could theoretically limit the rise of sociopolitical complexity (45, 46). Although a cold climate also prevents the cultivation of kava, we did not control for this variable, as it only applies to one case in our sample (the Māori in New Zealand). Similarly, we did not control for agriculture, as it is practiced by all societies in the sample. Assuming that models include all relevant confounders and that kava is more likely to lead to sociopolitical complexity than vice versa, the results should demonstrate the magnitude of kava’s causal effect, if any, as well as the influence of each confounder.

To examine the directionality and strength of the potential coevolution between kava and both sociopolitical traits, we employed a recently developed Bayesian method for generalized dynamic phylogenetic models (47, 48). This method models the coevolution of traits on a phylogenetic tree—in our case, the coevolution of cultural traits on a phylogeny of Austronesian languages (39)—allowing inferences of directionality and Granger causality (49) over evolutionary time.

## Results

### Bayesian Regression Models.

**Fig. 2.**
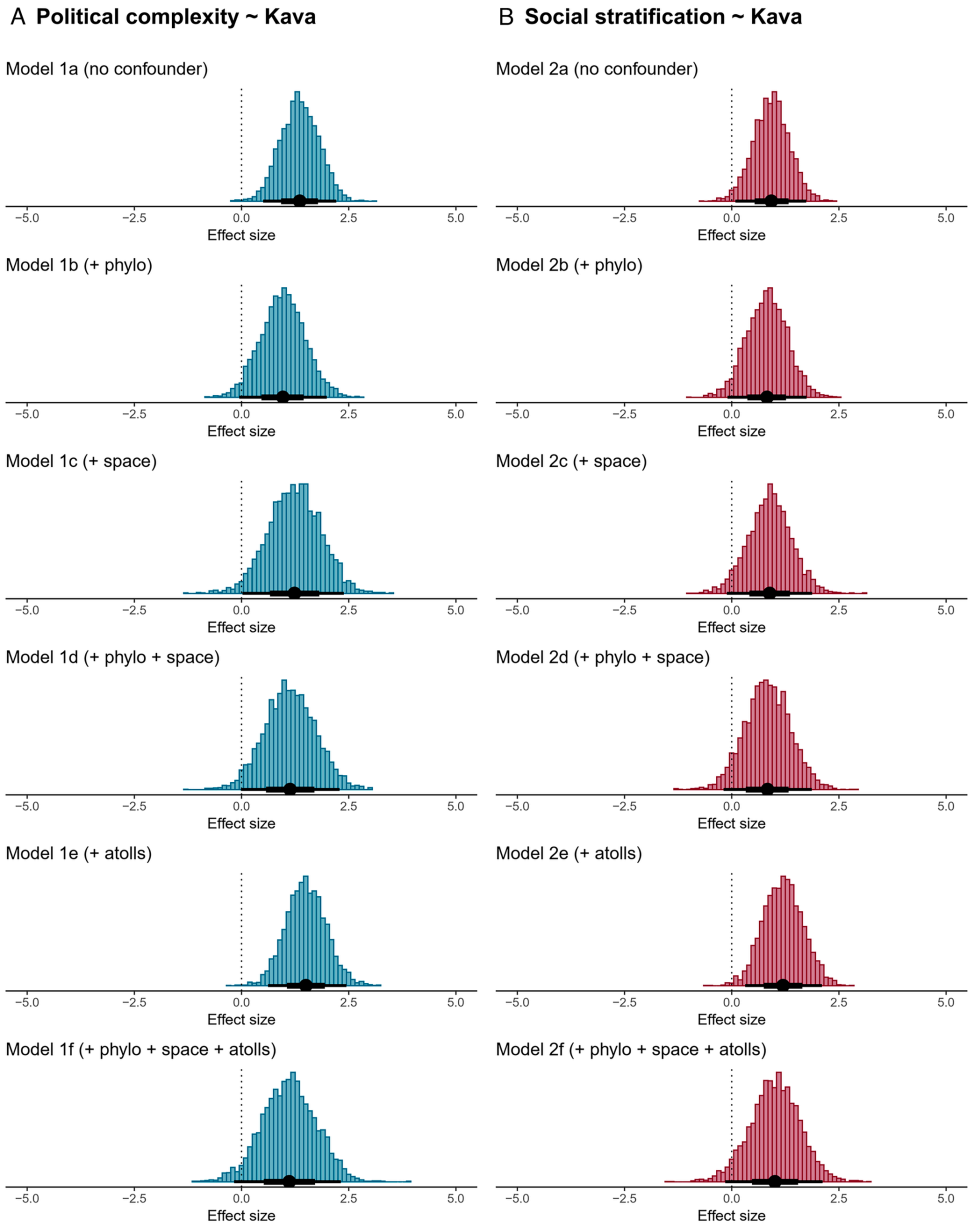
Posterior coefficient estimates of the associations between kava and two sociopolitical traits (political complexity and social stratification). All Bayesian regression models consistently show a positive correlation between kava and political complexity (*A*), as well as social stratification (*B*). However, effect sizes vary based on the structure of potential confounders (indicated in brackets), with some credible intervals including zero. Points and intervals show the mean and 66% and 95% credible intervals of each posterior distribution.

To evaluate the effect of kava on political complexity and social stratification, we ran several Bayesian ordinal regression models, each with a different set of potential confounders (Fig. 2). The first pair of models assumes no confounders (a). In contrast, the others account for phylogenetic relatedness (b), spatial proximity (c), location on atolls (e), and their combinations (d and f).

In our initial models without controls, we found positive associations between kava drinking and political complexity (mean log-odds slope = 1.36, 95% credible interval [0.51, 2.21]) and between kava drinking and social stratification (mean log-odds slope = 0.92, 95% CI [0.08, 1.74]). On the original five-point scales, these slopes imply an increase of 0.73 units in political complexity (95% CI [0.29, 1.17]) and an increase of 0.44 units in social stratification (95% CI [0.04, 0.83]) in kava drinking societies compared to societies in which kava drinking is absent. The magnitude of these effects is thus relatively modest, comparable to the impact of low-alcoholic fermented beverages on political complexity observed in a global cross-cultural study (3).

We found that the effect sizes varied when we accounted for confounders (Fig. 2 and *SI Appendix*, Tables S1 and S2; for detailed summaries of all analyzed models, see output folder at https://doi.org/10.5281/zenodo.15743660). When controlling for phylogenetic relatedness and spatial proximity among the societies in our sample, the effect sizes reduced and the credible intervals included zero. When controlling for atolls, the effect sizes increased slightly. To understand this increase, we ran additional analyses of the association between atolls and cultural traits (*SI Appendix*, Fig. S2), which revealed a substantial adverse effect of atolls on kava (as expected), but zero effect of atolls on social stratification and, unexpectedly, a positive relationship between atolls and political complexity. This finding suggests that atolls are unlikely to be a confounder in the positive associations between kava and either of the two sociopolitical traits.

Kava drinking, and to some extent also political complexity and social stratification, evince spatial clustering (Fig. 1 and *SI Appendix*, Fig. S1). Since there is no definitive way to control for spatial nonindependence (44), we conducted a sensitivity analysis to assess the robustness of our spatial control. Following Skirgård et al. (50), we ran several models (*SI Appendix*, Fig. S3 and Table S1), each with different parameterizations of a Matérn covariance function that quantifies the degree of dependence among societies based on their spatial distance (*SI Appendix*, Fig. S4). These parameterizations are based on the assumption that regular contact between Pacific societies occurred within 100 miles, i.e., an overnight voyage in traditional craft (51), while at greater distances the intensity of contact decreased exponentially. The results indicate that increasing the reach of spatial relationships reduces the effect size of kava on both sociopolitical variables, such that the 95% credible intervals for both variables encompass zero (*SI Appendix*, Fig. S3). Although the assumptions of some models may be exaggerated (e.g., medium to high covariance even at distances of several hundred kilometers), this sensitivity to the chosen covariance function parameters must be considered when interpreting the results.

### Testing the Coevolution of Kava and Sociopolitical Traits.

Our regression analyses revealed positive associations among kava, political complexity, and social stratification, with varying robustness to control variables. However, it remains unclear whether and how kava and sociopolitical traits have coevolved with over the evolutionary history of Oceanic societies.

As a first step in assessing coevolution, we examined the phylogenetic correlations and signals for kava drinking and both sociopolitical traits within the Austronesian language phylogeny. We found that kava was positively phylogenetically correlated with political complexity (phylogenetic correlation = 0.46, 95% CI [−0.01, 0.84]; *SI Appendix*, Fig. S5) and social stratification (phylogenetic correlation = 0.33, 95% CI [−0.35, 0.83]; *SI Appendix*, Fig. S6). However, there was uncertainty in these estimates, with credible intervals including zero. Moreover, while we found relatively strong phylogenetic signal for kava drinking (*ƛ* = 0.50, 95% CI [0.11, 0.82]), we saw weaker phylogenetic signal for political complexity (*ƛ* = 0.37, 95% CI [0.11, 0.64]) and social stratification (*ƛ* = 0.13, 95% CI [0.00, 0.37]; *SI Appendix*, Fig. S7). Together, these results suggest that while there is some tentative evidence of positive correlated evolution for kava drinking and sociopolitical traits, the correlations are uncertain. The distribution of political complexity and social stratification is not strongly explained by shared evolutionary history.

Building on these results, we used generalized dynamic phylogenetic models to infer potential directionalities in the coevolution of kava drinking and sociopolitical traits. While previous methods for testing the coevolution of traits have been restricted to binary variables only (52), here we assume that the kava drinking variable (binary) and the sociopolitical variables (ordinal) represent latent continuous variables that are allowed to coevolve along the branches of the phylogenetic tree. This method enables us to infer causal directionalities and contingencies in the cultural evolution of these traits (see *Methods* for more details).

**Fig. 3.**
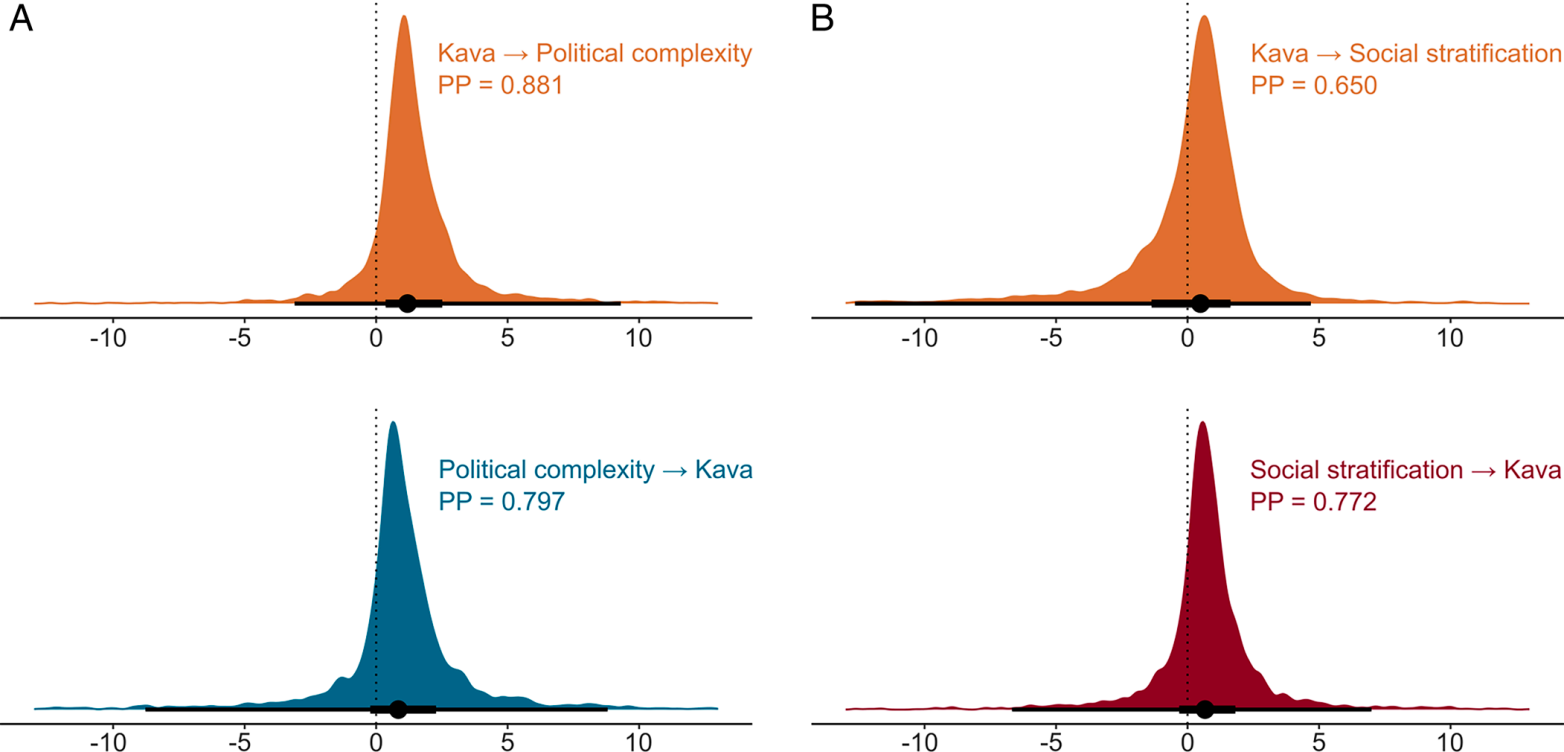
Weakly positive yet highly uncertain evidence for coevolutionary relationships between kava drinking and the sociopolitical traits in models without controls for spatial autocorrelation (i.e., Model 0 in *SI Appendix*, Table S3). Posterior distributions showing probability densities of changes in the equilibrium trait value θ of (*A*) kava and political complexity, and (*B*) kava and social stratification, in response to a standardized unit increase in the other trait. Posterior probabilities (PPs) denote the positive posterior mass (that is, the probability) that the presence of kava leads to an increase in political complexity, or social stratification, and vice versa. The values were scaled by the median absolute deviation, which is less sensitive to outliers than the standard deviation. Points and intervals show the median and 66% and 95% credible intervals of each posterior distribution.

When we fitted these dynamic phylogenetic models to the data, we found only weak evidence for coevolutionary relationships between kava drinking and the sociopolitical traits. Fig. 3 shows the posterior change in the equilibrium values for each trait given a standardized increase in the other trait (Δθ). For the model that included kava drinking and political complexity, we found slightly positive yet highly uncertain effects, both from kava drinking to political complexity (median Δθ = 1.19, 95% CI [−3.10; 9.30], positive posterior mass = 88%) and vice versa (median Δθ = 0.84, 95% CI [−8.77; 8.80], PPM = 80%). For the model that included kava drinking and social stratification, we found a similar pattern, with weakly positive and highly uncertain effects both from kava drinking to social stratification (median Δθ = 0.49, 95% CI [−12.64; 4.69], PPM = 65%) and vice versa (median Δθ = 0.67, 95% CI [−6.68; 6.99], PPM = 77%).

**Fig. 4.**
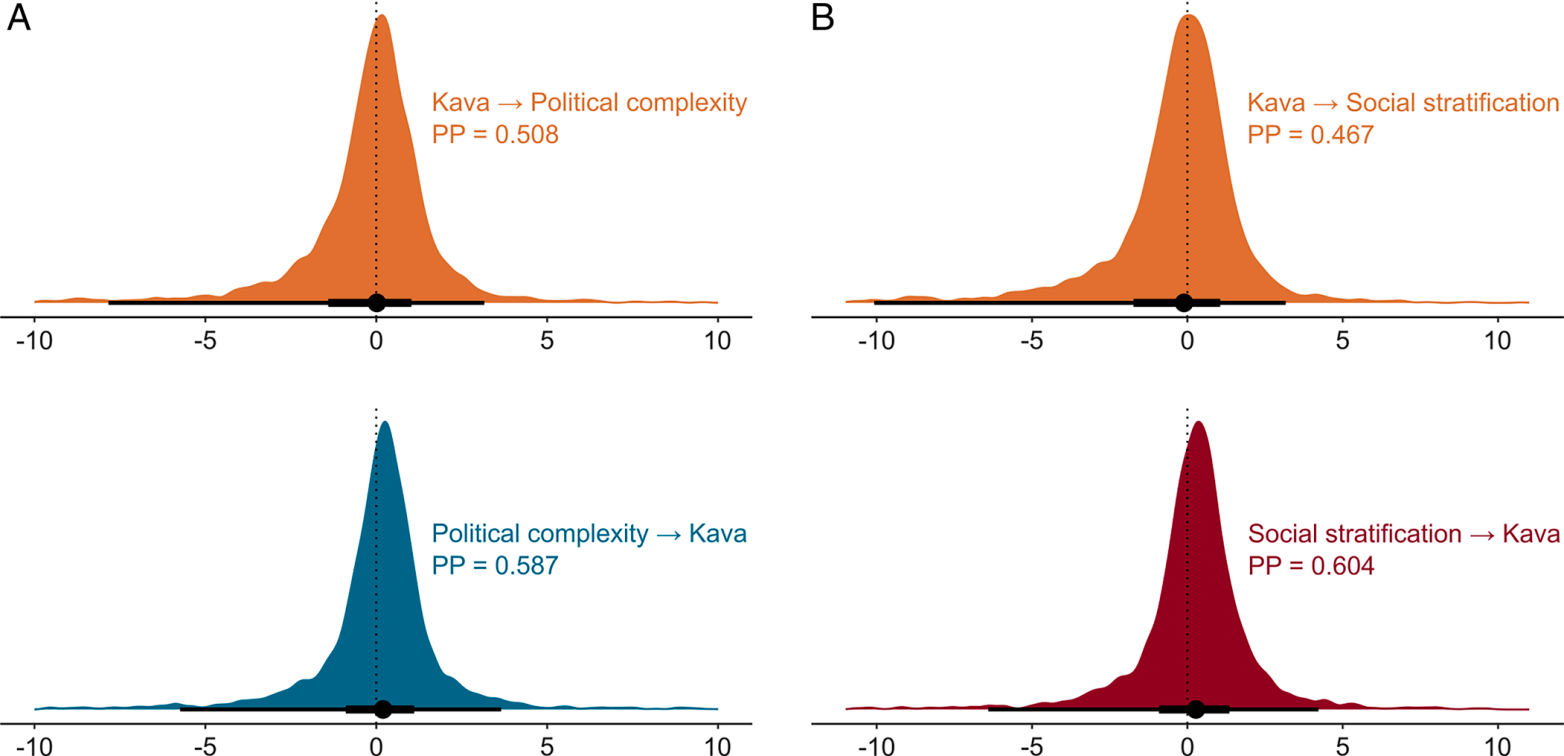
No evidence of coevolutionary relationships between kava drinking and political complexity (*A*) or social stratification (*B*) in models controlling for spatial autocorrelation (i.e., Model 1 in *SI Appendix*, Table S3). For a detailed description of the graphs, see the caption for Fig. 3.

When we additionally controlled for spatial autocorrelation in these dynamic phylogenetic models, we found no evidence of coevolutionary relationships between kava drinking and sociopolitical traits (Fig. 4 and *SI Appendix*, Table S3). Regardless of the prior we used for the spatial nonindependence controls, we found that the coevolutionary effects straddled zero for both political complexity and social stratification.

## Discussion

Kava is widely consumed in Oceania, yet its potential role in the evolution of sociopolitical complexity in this region has remained unclear. We studied the associations between kava consumption, political complexity, and levels of social stratification across 83 Oceanic-speaking societies. Our results were mixed. While we initially found positive relationships between kava consumption and sociopolitical traits, these relationships were not robust to controlling for spatial and cultural phylogenetic nonindependence between societies. We also found no evidence for coevolution between kava consumption and sociopolitical traits after controlling for spatial nonindependence. Taken together, these results suggest that kava was not one of the major factors contributing to the rise of sociopolitical complexity in Oceania.

Several examples illustrate the limited importance of kava for cultural complexity. Kava drinking societies on Vanuatu, a presumed region of its domestication, show only low and medium levels of sociopolitical complexity, as measured by our two variables (Fig. 1 and *SI Appendix*, Fig. S1). Societies on the Santa Cruz Islands and Taumako also had low levels of complexity, despite the occasional use of kava (23, 26). The same applies to rare cases of societies consuming kava in New Guinea, both in the area around Madang and on the southern coast, including non-Oceanic-speaking societies such as the Marind-anim, Kiwai, and Keraki (26). The introduction of kava, albeit relatively recent (23), has not led to an increase in their sociopolitical complexity (53). On the other hand, several societies have developed high levels of complexity without using kava, specifically in New Caledonia and the Marshall Islands.

However, much more important than its mere presence may be the intensity with which kava is incorporated into social life. Indeed, the most developed “kava complex” is found in mid- to high-complex societies such as Fiji, Tonga, Samoa, Rotuma, Futuna, Uvea, and Pohnpei (32, 54). There, kava is consumed regularly in various contexts, including ritual, political ceremonies, and informal gatherings. In contrast, people on the Santa Cruz Islands consumed kava only on special occasions such as funerals (55, 56). Unfortunately, ethnographies do not provide sufficient data on the frequency and intensity of kava consumption for most societies, making it difficult to construct ordinal measures to test this conjecture.

Moreover, most societies with an elaborated kava culture and a “shared ritual format” (57) are from western Polynesia, strongly influenced by the Tongan maritime expansion during the late prehistoric and early historic periods (58). Such close geographical and historical links between societies are the reason why we controlled for spatial autocorrelation in the analyses. However, these connections simultaneously obscure whether kava could have led to the rise of sociopolitical complexity, or whether, on the contrary, existing hierarchies could have led to a stronger integration of kava into social and political life. For instance, it is possible that Polynesian elites utilized kava rituals in response to increased political competition following European contact, thereby strengthening and legitimizing their rule, which led to greater intensification and formalization of kava consumption (23). Fiji provides an interesting example in this regard (33, 34, 57, 59). In precontact times, kava originally had a predominantly religious function. Priests used strong infusions of kava to induce shamanic trances, enabling them to communicate with ancestors and deities. Kava consumption was restricted to priests, chiefs, and elders. Women were excluded from access to supernatural spirits, while low-ranking men were allowed only to prepare and serve kava to high-ranking men. It was only later (in the 18th century under Tongan influence) that political leaders took over the kava ceremony and its function became more sociopolitical. Christianity later relaxed restrictions on drinking for women and young men, and kava drinking became an integral part of daily village life, consumed also in many recreational and informal settings.

The extent to which the Fijian local history of kava’s functions can be generalized is uncertain, given the wide range of ethnographically documented kava use among Oceanic peoples and the absence of deep historical records. Nevertheless, it appears that kava’s association with the sacred and death, as well as its traditional use as a medium of communication with supernatural spirits, was widespread (27). For example, on Tikopia, kava was commonly poured as a libation to the gods but rarely consumed; when consumed, it was mainly by spirit media in a trance (26). Yet, the religious and ritual role of kava does not, in itself, imply a strict separation from politics. As a recent study has shown (48), religious and political authorities in Pacific societies were closely intertwined and probably coevolved. Spiritual power is often necessary to legitimize political power. As documented in many societies’ kava ceremonies, chiefs and title holders not only communicate with their ancestors and deities but also become their temporary reincarnations and living manifestations (22, 57). Through access to the supernatural power, they fulfill their leadership role in securing prosperity and general welfare.

### Kava versus Alcohol.

Slingerland (2) has suggested that the “drunk hypothesis” could apply also to kava, as it has many social effects similar to alcohol. These include increasing sociability, cooperation, and group bonding, especially among men, as well as reducing stress and anxiety, including that from crowded conditions and hierarchical inequalities. Both intoxicants also play an important role in communicating with ancestors and gods, as potential truth-telling technologies and sources of creative insights. Compared to psychedelics, both can also be relatively easily incorporated into everyday social life.

Nevertheless, some aspects are different. While kava consumption usually leads to a state of calmness and tranquility, alcohol inebriation more often leads to ecstatic group experiences, especially when combined with music and dance. From a sociopolitical perspective, both intoxicants have been used ceremonially and ritually to confirm and reinforce existing hierarchies. However, alcohol was frequently used to acquire political power as part of competitive feasting (60) or by mobilizing labor through work-party feasts (61), both of which may have contributed to increased political complexity. In hierarchical societies, its production was often centralized and redistributed from the elites to the nonelites, including its use as compensation for large community projects, such as building monumental architecture or maintaining irrigation canals. As far as we know, none of this was typical in the case of kava.

Fermented alcoholic beverages also have significant caloric value, which kava lacks. Some argue that it was precisely this combination of nutritional and psychoactive effects that led to the initial cultivation and domestication of many fermentable crops, especially cereals (2, 60). Thus, alcohol could have contributed to greater cultural complexity also indirectly—through its potential role in the emergence of agriculture. In contrast, kava was domesticated by societies that were already agricultural, cultivating crops such as taro and yams (23, 62), making this causal pathway inapplicable.

Overall, it seems that in Oceania, in the absence of alcohol, factors other than kava played a greater role in the rise of sociopolitical complexity (for an overview, see ref. 62). These may have included environmental and geographic conditions (45, 46, 58), high population density enabled in Remote Oceania by the absence of malaria and other diseases (62), economic intensification and specialization (38), rapid population rise caused by in-migration (63, 64), increased intergroup conflict resulting from climatic cooling and drying (65), the development of religious hierarchies (48), and ritual human sacrifice (66).

### Limitations and Conclusion.

Our study relies on language phylogenies, which generally reflect population and cultural history well (67). However, as genomic research has shown (68), a rare population replacement with language continuity occurred in Vanuatu 2,500 y ago. Such contact could have influenced the evolution of political organization (e.g., the introduction of lower political complexity in Vanuatu), which our coevolutionary models cannot account for. On the other hand, kava may have only recently played a sociopolitical role in Oceania, as illustrated by the Fijian example. If this were true, coevolutionary models would also struggle to detect it, as they assess effects across the entire phylogenetic tree. The null result of coevolutionary relationships may also be due to the low statistical power. Although we collected data on as many societies as possible, models with larger sample sizes could have provided more substantial evidence of coevolutionary effects. However, their magnitude would likely remain small nonetheless. Similarly limiting is the use of a binary kava presence variable instead of quantitative data on the intensity of its consumption, which, unfortunately, is not available. Finally, the results may be obscured by the strong mutual interdependence of Oceanic societies. Although it is crucial to control for spatial and cultural nonindependence (44), without detailed historical knowledge, it is difficult to disentangle the specific roles of horizontal versus vertical transmission in the spread of kava and sociopolitical institutions. The uncertainty in this prior knowledge, combined with that of the phylogenetic tree, yields varying results from coevolutionary models (Figs. 3 and 4 and *SI Appendix*, Tables S3–S6).

Ethnographic evidence shows that kava played (and in many Pacific societies still plays) a significant role in sacralizing social hierarchy and inequality through its spiritual value, ritualized consumption, and traditional restrictions based on age, gender, and rank (69). Kava rituals served to separate men and women, as well as the young and the old, and to honor chiefs and other high-status individuals, helping to maintain and strengthen the existing political and social order (23). However, our results suggest that kava consumption was unlikely to have been a major driver of sociopolitical complexity, thereby challenging the maximalist version of the “drunk hypothesis.”

## Materials and Methods

### Coding of Variables.

We collected data on four variables—kava drinking, political complexity, social stratification, and location on atolls—for a sample of 83 Oceanic-speaking societies, as they were immediately before the colonial period. We coded kava drinking in binary form: 0 if it was absent in the society and 1 if it was present. The coding of kava was based on information in Brunton (26) and Lebot et al. (23); however, when in doubt, we also consulted primary ethnographic sources. In five cases, however, information was insufficient or conflicting (coded NA). We note that uncertainty regarding precontact kava consumption was particularly prevalent in societies located on the borders of the Solomon Islands and Vanuatu, such as Nendo, Main Reef Island, Vanikoro, Utupua, Anuta, Tikopia, the Banks Islands, and Malaita Island. As a proxy for sociopolitical complexity, we chose two five-point ordinal variables—political complexity and social stratification—both originally created by Murdock and Provost (37). The former measures the number of distinct jurisdictional levels recognizable in the society, classifying societies on a scale from politically uncentralized even at the community level (coded 0) to societies with three or more administrative levels above the local community (coded 4). The latter measures the relative complexity of graded status distinctions within society, classifying societies on a scale from egalitarian (coded 0) to societies with three or more social classes or castes (coded 4). The coding of these variables was based on a range of ethnographic sources, which are listed together with the justification for our coding decisions in Dataset S1. The coding of the kava variable was performed by two coders (V.H. and O.S.), while political complexity and social stratification were coded by a single coder (O.S.) who specializes in this topic (48, 66, 70). We obtained data on political complexity for all 83 societies, but there is one missing value for social stratification (namely Lifou). We coded societies living on atolls as 1 and those not living on atolls as 0. In seven cases, we applied the code 0.5 for “almost atolls,” as these did not fit the strict definition of an atoll. Our codes were based on whether or not the island was listed in Goldberg (71). For summary cross-tables, see *SI Appendix*, Tables S7–S9.

### Phylogenies.

We modeled cultural ancestry using a sample of 100 randomly drawn trees from the posterior distribution of Austronesian language phylogenies, created by Gray et al. (39). For some analyses, we used their maximum clade credibility tree or “summary tree.” Both types of trees are available at https://doi.org/10.5281/zenodo.10149668 (72). The original set of trees included 400 languages, of which 83 corresponded to our database of Oceanic-speaking societies with adequate ethnographic information. Nine societies (Chuuk, Futuna, Lakalai, Marshall Islands, Nendö, Nissan, Tabar, Vanikoro, Ifaluk) corresponded to more than one language. In these cases, we chose only one language per society, the one that most closely matches the ethnographic record. We pruned the phylogenies using the *ape* package (73) in the programming language R (74).

### Bayesian Regression Models.

We ran Bayesian regression models using the R package *brms* (41). We modeled political complexity and social stratification as ordered factors (each with five different levels) and analyzed the effects of their potential predictors in ordinal regression models. We set the family argument in *brm*() models as *cumulative()*, assuming that the response variables originate from the categorization of latent continuous variables (75). In models where kava was a response variable (*SI Appendix*, Fig. S2), we changed this argument to *bernoulli()*. The kava and atolls were coded as binary factors (i.e., absence versus presence). We tried two ways of binarizing the atolls variable: one treated “almost-atolls” as NAs (i.e., excluded them), while the other treated them as atolls. However, the results were qualitatively the same. We used regularizing priors for all models to impose conservatism on parameter estimates.

To account for spatial autocorrelation and phylogenetic nonindependence (42–44), we included two correlation matrices in our models: one based on geographic distances and another based on historical relatedness. To control for spatial effects (i.e., horizontal transmission), we calculated Haversine (great-circle) distances between societies using their longitude and latitude coordinates. We normalized these distances to lie between 0 and 1 and then created a covariance matrix using the Matérn 3/2 kernel function. Given our uncertainty about the strength of spatial diffusion for these cultural traits throughout the history of Oceanic societies, we ran our models using four different curves for the decay in spatial covariance as a sensitivity analysis (*SI Appendix*, Figs. S3–S4), setting the parameter *rho* to 0.02, 0.04, 0.06, and 0.08. All curves assume strong covariance at short distances, such as regular cultural contact within a distance of an overnight voyage in traditional craft [i.e., about 100 miles; (51)], and subsequent exponential decline. However, they differ in the reach of less frequent contact. While the *rho* 0.02 assumes zero covariance beyond 1000 km, the other values allow for substantial covariance even at greater distances. As a control for shared ancestry (i.e., vertical transmission), we calculated phylogenetic distances between societies based on Gray et al.’s (39) Austronesian language phylogenies. We used languages as a proxy for cultural relatedness because they provide a good reflection of population and cultural history (67). After pruning the phylogeny to 83 societies in our sample, we used the *vcv.phylo* function in the *ape* package (73) to create a correlation matrix, setting the correlation parameter, *corr*, to true. We added the spatial and phylogenetic distance correlation matrices as group-level parameters (“random” effects) to the *brm()* models using the syntax *gr()*.

The models estimate a posterior distribution of possible coefficient estimates (i.e., the effect of the predictor on the response variable) on the log-odds scale. To understand the effects of kava on political complexity and social stratification on their original ordinal scale, we calculated model-based predictions using the *fitted()* function in the *brms* package (41). For each model in Fig. 2, we estimated the predicted average value of political complexity and social stratification in the absence and presence of kava drinking, i.e., by setting kava at 0 and 1, respectively. The difference between predicted values then indicates the expected average change in political complexity and social stratification levels on the original five-point scale when kava is present (*SI Appendix*, Table S2).

### Assessing Phylogenetic Correlations and Signals.

We assessed phylogenetic correlations and signals using Bayesian multivariate multilevel models. We included kava drinking as a binary outcome variable (Bernoulli response distribution, logit link) and either political complexity or social stratification as an ordinal outcome variable (cumulative response distribution, logit link). We included correlated random effects for Oceanic-speaking societies, allowing these random effects to covary according to the phylogenetic distances between societies. We used regularizing priors to impose conservatism on model estimates. These models were run using the *brms* R package (41), iterating the model over 100 randomly drawn posterior trees to account for phylogenetic uncertainty. The models converged normally. We calculated phylogenetic signals from the resulting models as the proportion of variation captured by phylogeny (76).

### Generalized Dynamic Phylogenetic Models.

Static phylogenetic correlations cannot tell us about directionalities in coevolution—whether evolutionary change in one trait precedes evolutionary change in another trait. To assess these directionalities and make claims about Granger causality on the phylogenetic tree, we fitted generalized dynamic phylogenetic models to the data. Unlike approaches that require users to dichotomize ordinal traits (52), generalized dynamic phylogenetic models allow users to model traits of any distribution as latent variables that coevolve over the branches of the phylogeny.

The observational model for societies *i* is as follows:

$${\text{kava}}_{i}∼\text{Bernoulli}\left(p_{i}\right),$$

$${\text{complexity}}_{i}∼\text{Ordered}\left(\phi_{i},\alpha \right),$$

$$\text{logit}\left(p_{i}\right)=\eta_{1,i},$$

$$\text{logit}\left(\phi_{i}\right)=\eta_{2,i},$$

$$\alpha ∼N\left(0,2\right),$$

where α is a vector of ordered cutpoints and *η* represents the latent coevolving variables for both traits at the tips of the phylogeny. The latent evolutionary model is as follows:

$$d\eta \left(t\right)=\text{b}+\text{A}\eta \left(t\right)+\text{G}dW\left(t\right),$$

$${\text{GG}}^{\prime }=Q=\left(\begin{array}{cc} \sigma_{1} & 0\\ 0 & \sigma_{2} \end{array}\right)\text{R}\left(\begin{array}{cc} \sigma_{1} & 0\\ 0 & \sigma_{2} \end{array}\right),$$

$${\text{b}}_{1},{\text{b}}_{2}∼N\left(0,1\right),$$

$${\text{A}}_{1,1},{\text{A}}_{2,2}∼N^{-}\left(0,1\right),$$

$${\text{A}}_{1,2},{\text{A}}_{2,1}∼N\left(0,2\right),$$

$$dW\left(t\right)∼\sqrt{\mathrm{dt}}N\left(0,1\right),$$

$$\sigma_{1},\sigma_{2}∼N^{+}\left(0,1\right),$$

$$\text{R}∼\text{LKJcorr}\left(4\right).$$

We model trait evolution as a stochastic differential equation that unfolds along the branches of the Austronesian language phylogeny. Coevolution of latent variables follows a multivariate Ornstein–Uhlenbeck process, with components for selection (the 2 × 2 *A* matrix) and exogenous drift (the 2 × 2 *G* matrix). Diagonals of the A matrix represent autoregressive selection (the effect of a trait upon itself) and the off-diagonals represent cross-trait selection (the effect of a trait upon the other trait). We used the off-diagonals from the *A* matrix to derive standardized measures of the strength of coevolution between traits (Δθ, Figs. 3 and 4). The *G* matrix, which scales the Wiener drift process [*W*(*t*)], is the Cholesky decomposition of the *Q* covariance matrix for exogenous correlated drift. The *b* vector represents the continuous-time intercepts for each trait.

To incorporate controls for spatial autocorrelation into this model, we also included a Gaussian process in the linear observational model for each trait, following a Matérn 3/2 covariance function. The Gaussian Processes allowed varying intercepts for each society to covary according to the Haversine distances between societies.

Because it is likely that Proto-Oceanic societies did not use kava (23) and their political complexity was relatively low (36), we adjusted the prior on the tree root accordingly, setting the *eta_anc* parameter to *normal* (−2, 1). For a comparison of the results with the uninformed default prior, i.e., *std_normal()*, see *SI Appendix*, Tables S3–S6.

We fitted these models to the data using the recently developed *coevolve* R package (https://github.com/ScottClaessens/coevolve; v0.1.0) and Stan v2.35 (77). We iterated the model over 100 randomly drawn posterior trees to account for phylogenetic uncertainty. The models converged normally.

## Supplementary Material

Appendix 01 (PDF)

Dataset S01 (XLSX)

## Data Availability

Dataset on kava drinking, political complexity, social stratification, and location on atolls is provided with this paper as Dataset S1. Gray et al.’s (39) Austronesian language phylogenies are available at https://doi.org/10.5281/zenodo.10149668 (72). All data and code used for this study are also available at https://doi.org/10.5281/zenodo.15743660. All other data are included in the manuscript and/or supporting information.
