# Supplementary material for: Kava consumption and the rise of sociopolitical complexity in Oceania
Source: Proc Natl Acad Sci U S A. 2026 Feb 23;123(9):e2521658123. doi: 10.1073/pnas.2521658123 (PMC12956823; doi:10.1073/pnas.2521658123)
Supplement: Supplementary file 1 — Appendix 01 (PDF) [file pnas.2521658123.sapp.pdf]

PNAS

Supporting Information for
Kava consumption and the rise of sociopolitical complexity in Oceania

Václav Hrnčíř, Oliver Sheehan, Scott Claessens, Russell D. Gray

*Václav Hrnčíř
Email: vaclav_hrncir@eva.mpg.de

This PDF file includes:

Figures S1 to S7
Tables S1 to S9
Readme for Dataset S1
Full References for Dataset S1
SI References

Other supporting materials for this manuscript include the following:

Dataset S1

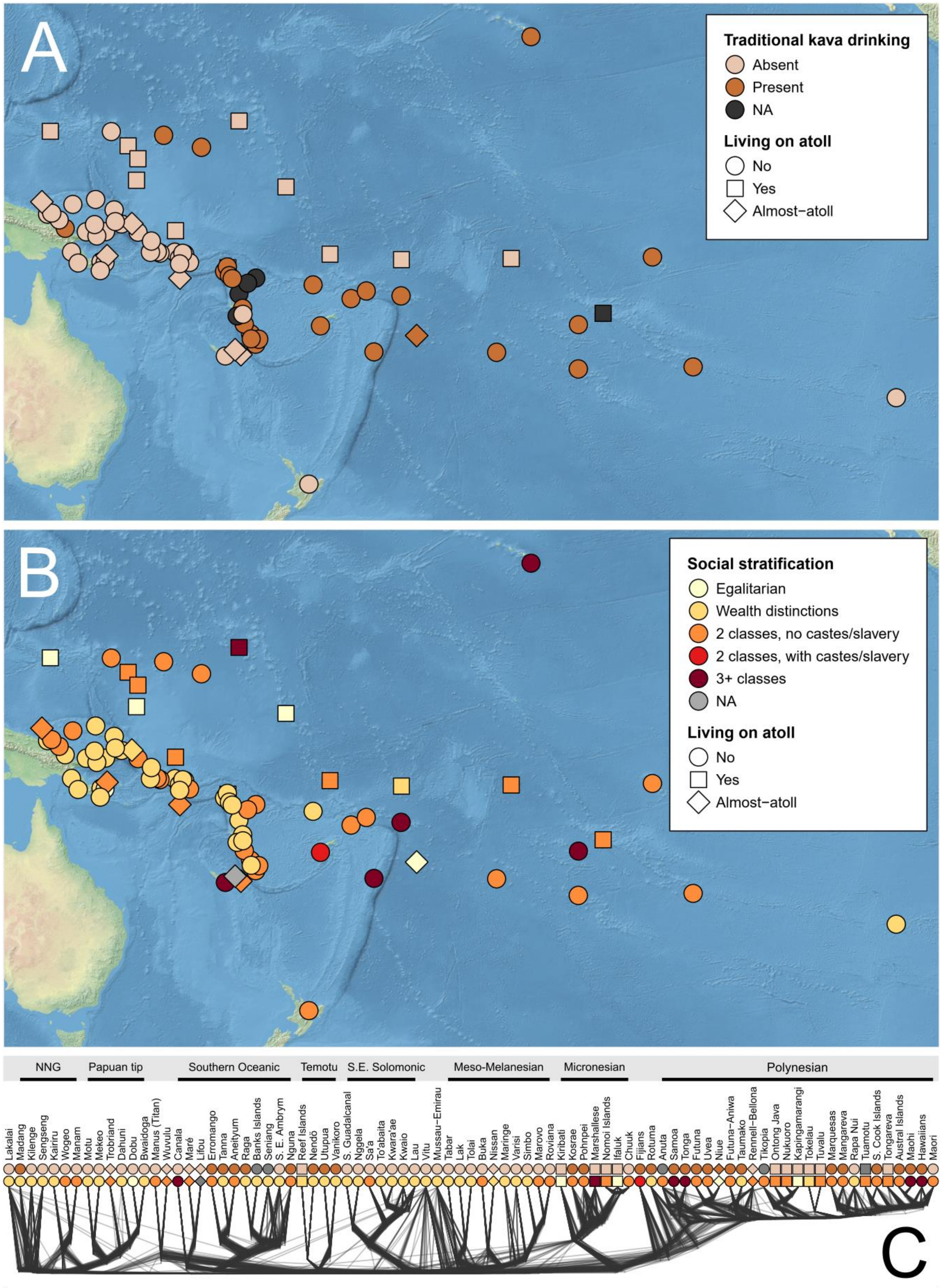

Fig. S1. Distribution of (A) traditional kava drinking and (B) social stratification in 83 Oceanic-speaking societies. Panel (C) shows both cultural traits plotted on the language phylogeny based on 100 randomly drawn posterior trees from Gray et al. (1). NNG, North New Guinea linkage. (Images created using map data from Natural Earth, www.naturalearthdata.com).

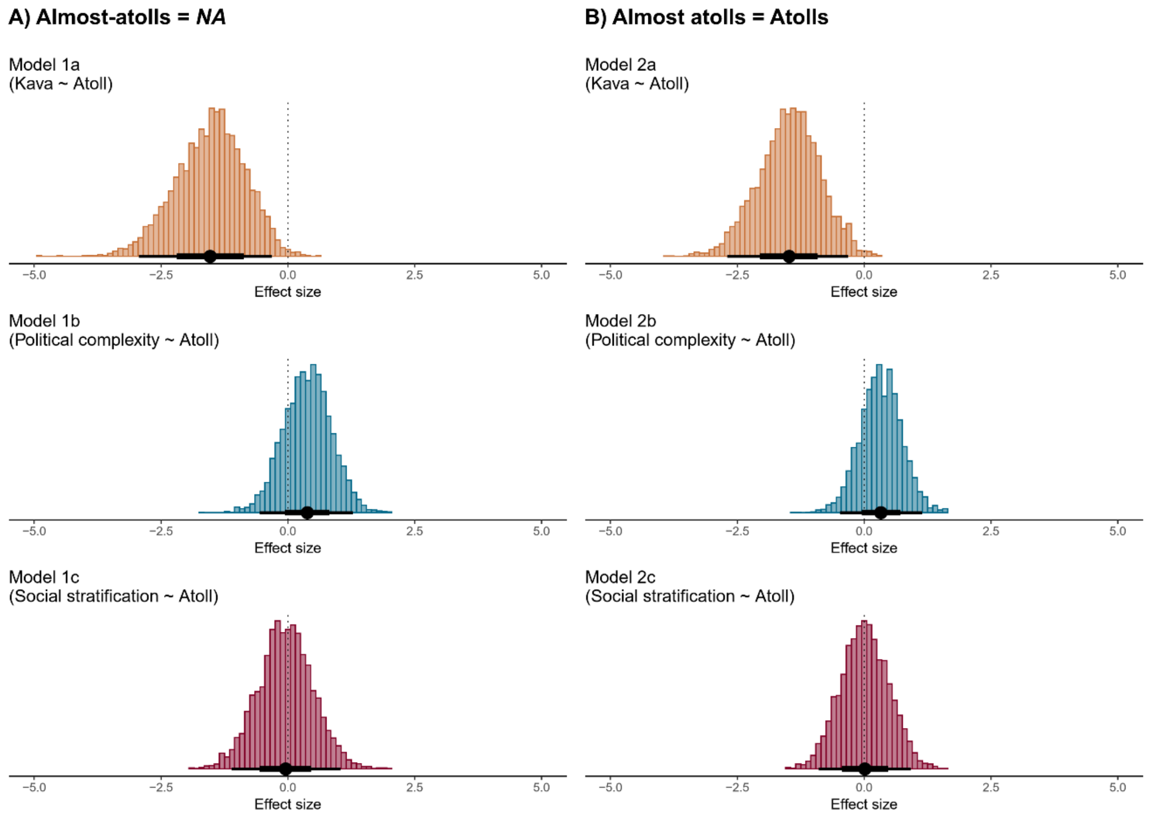

Fig. S2. Posterior coefficient estimates of the association between atolls and individual cultural traits (kava, political complexity and social stratification). Bayesian regression models show a strong negative correlation between atolls and kava (top panel), while the association between atolls and both sociopolitical variables are weaker or nonexistent (middle and bottom panels). The left panel (A) shows models where cases of “almost-atolls” are excluded. The right panel (B) shows models where almost-atolls are treated as atolls. Points and intervals show the median and 66% and 95% credible intervals of each posterior distribution.

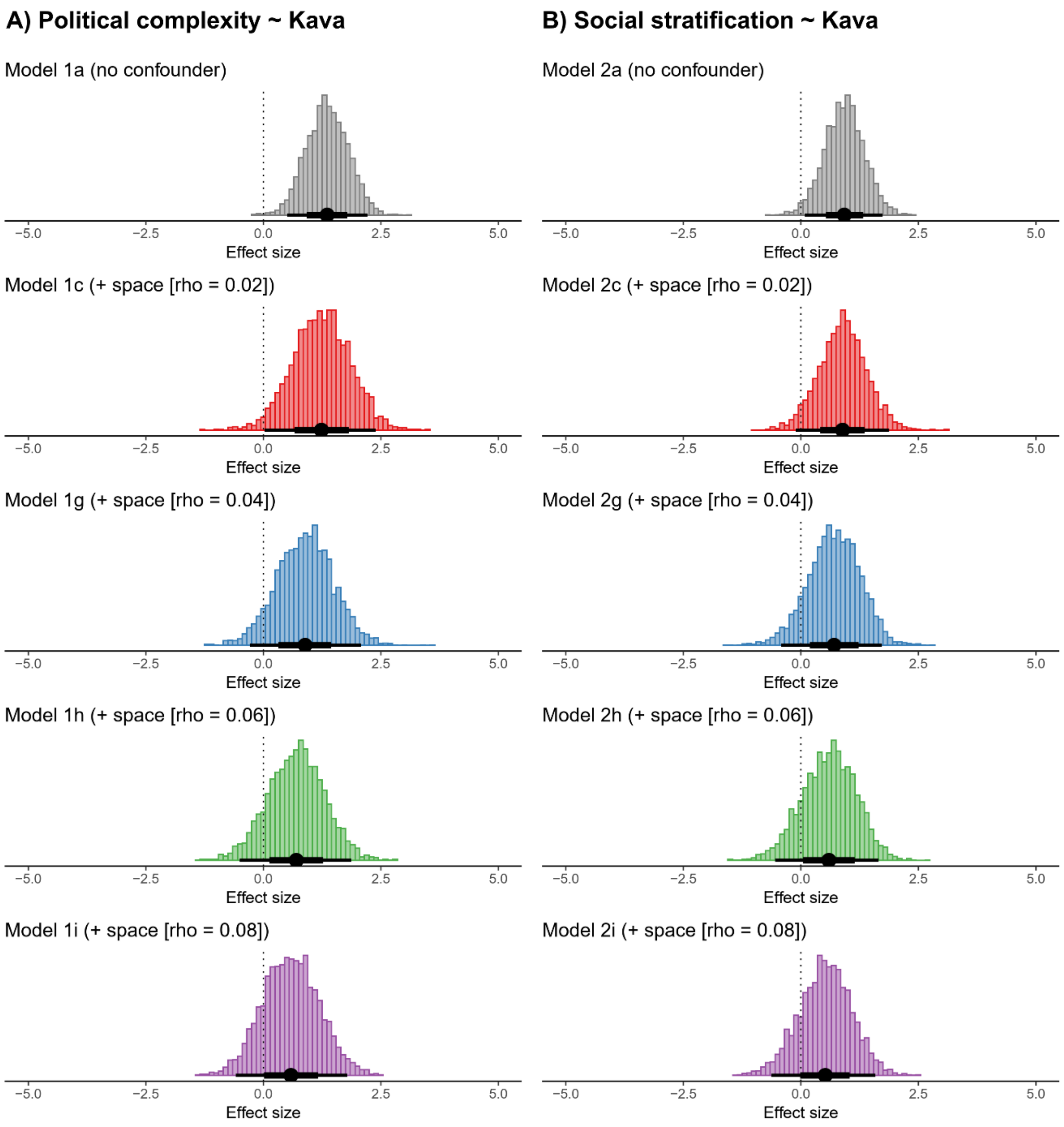

Fig. S3. A comparison of the effects of kava on political complexity (left panel) and social stratification (right panel) in four Bayesian regression sensitivity runs. We used different curves for the decay in dependence among societies with the spatial distance between them, based on various parameterisations of a Matérn covariance function (see Fig. S4). Effect sizes vary substantially depending on the chosen rho parameter (indicated in brackets); the higher the rho the lower the effect size. Points and intervals show the median and 66% and 95% credible intervals of each posterior distribution.

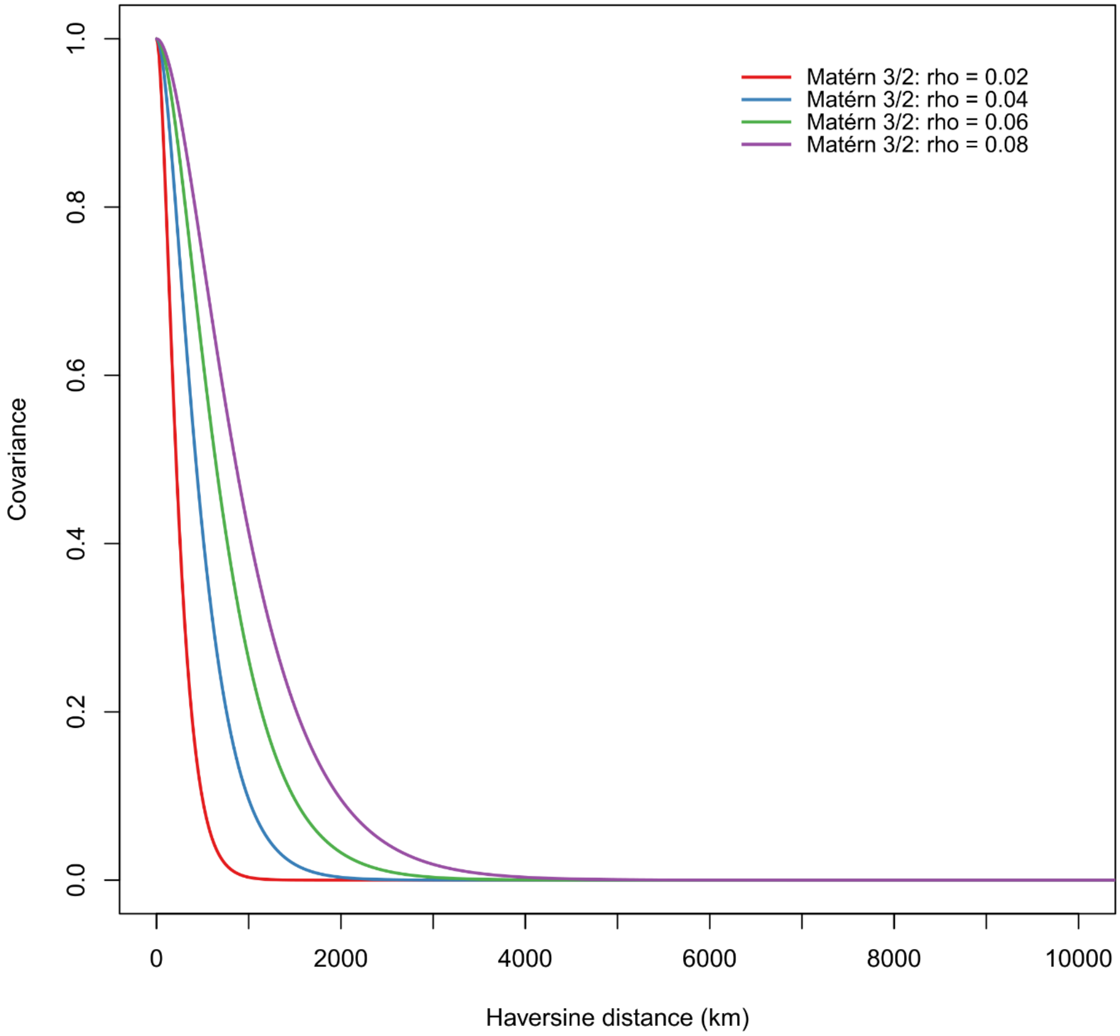

Fig. S4. The relative decay in spatial covariance based on the various parameterisations of a Matérn covariance function. Rho parameters (and the colors) correspond to those used in the sensitivity runs in Figure S3. Figure adapted after Skirgård et al. (2).

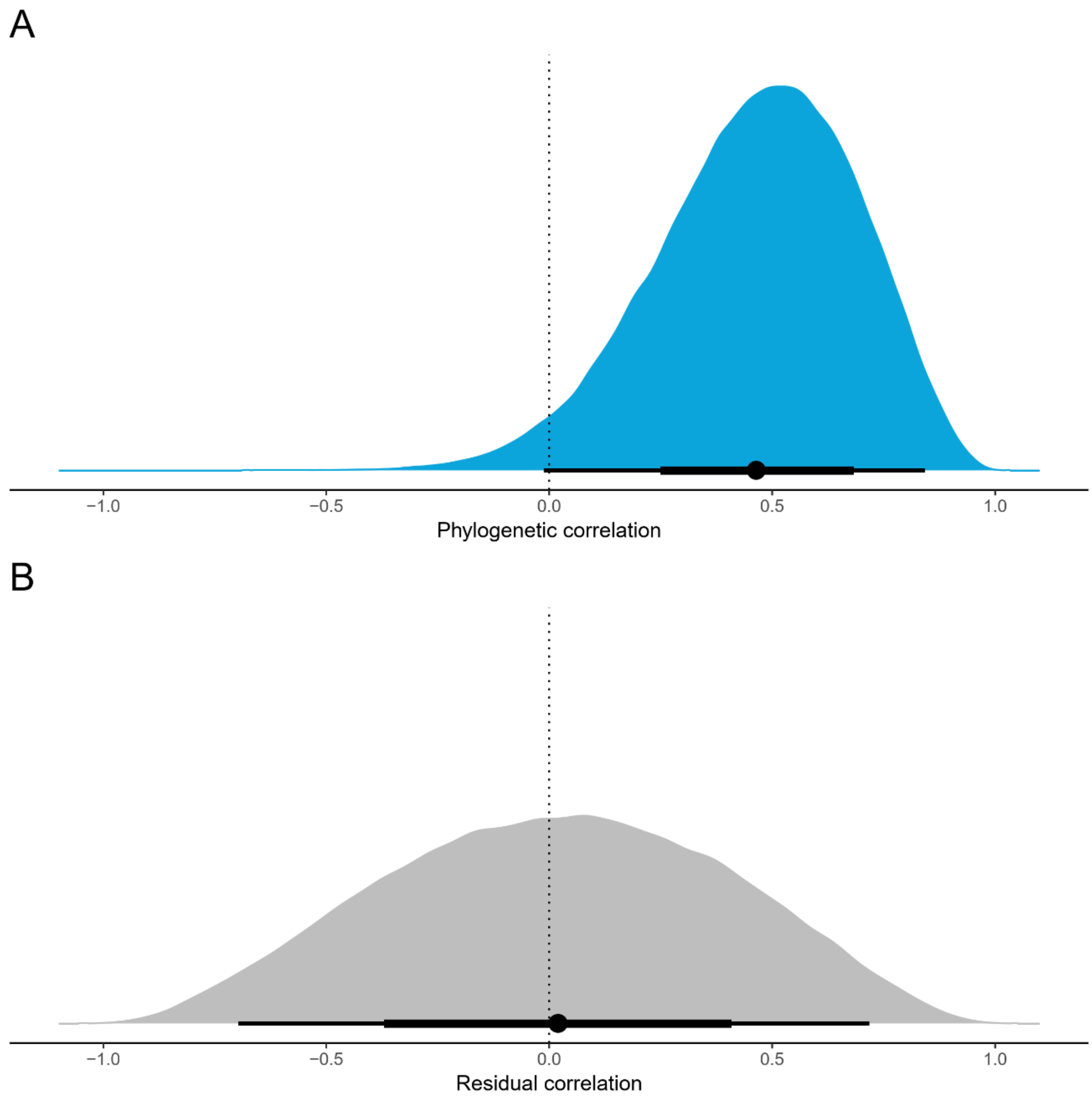

Fig. S5. (A) Phylogenetic and (B) residual correlations between kava and political complexity, estimated simultaneously in a Bayesian phylogenetic generalised linear mixed model. Points and intervals show the median and 66% and 95% credible intervals of each posterior distribution.

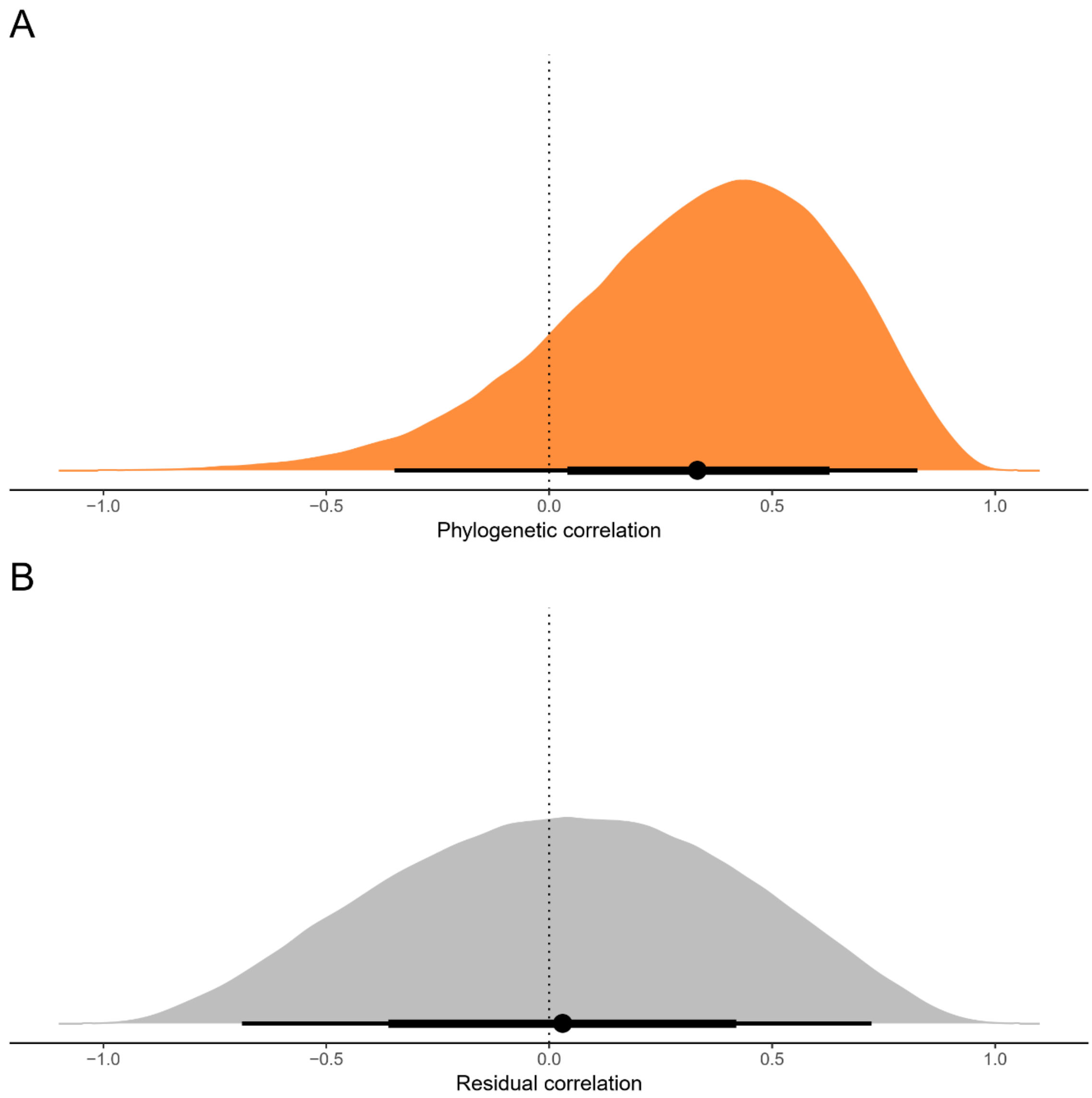

Fig. S6. (A) Phylogenetic and (B) residual correlations between kava and social stratification, estimated simultaneously in a Bayesian phylogenetic generalised linear mixed model. Points and intervals show the median and 66% and 95% credible intervals of each posterior distribution.

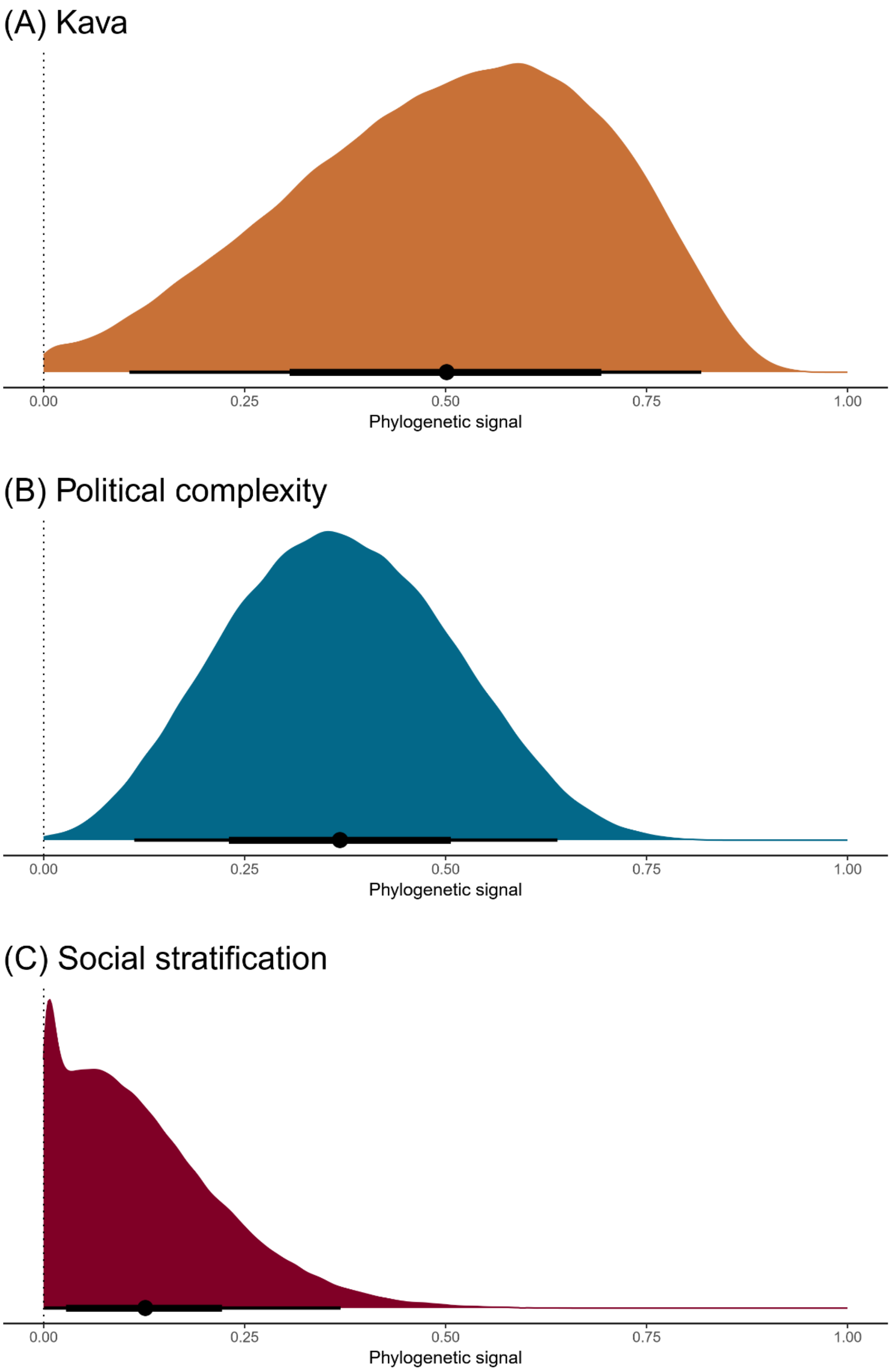

Fig. S7. Phylogenetic signal for (A) kava, (B) political complexity and (C) social stratification, as estimated by a Bayesian phylogenetic generalised linear mixed model. Points and intervals show the median and 66% and 95% credible intervals of each posterior distribution.

Table S1. The effect of kava on political complexity and social stratification based on the Bayesian ordinal regression models. All models show a positive correlation between kava and both sociopolitical variables, however, effect sizes vary based on the structure of potential confounders, with some credible intervals including zero. Coefficients are on the log-odds scale.

Model	*N*	Mean	2.5% CI	97.5% CI
**Political complexity**				
**Model 1a**<br>no confounders	78	1.36	0.51	2.21
**Model 1b**<br>+ phylo	78	0.97	-0.05	1.99
**Model 1c**<br>+ space [rho = 0.02]	78	1.24	0.03	2.39
**Model 1d**<br>+ phylo + space [rho = 0.02]	78	1.13	-0.01	2.29
**Model 1e**<br>+ atolls	71	1.50	0.62	2.45
**Model 1f**<br>+ atolls + phylo + space [rho = 0.02]	71	1.11	-0.17	2.32
**Model 1g**<br>+ space [rho = 0.04]	78	0.89	-0.29	2.08
**Model 1h**<br>+ space [rho = 0.06]	78	0.70	-0.50	1.87
**Model 1i**<br>+ space [rho = 0.08]	78	0.59	-0.58	1.79
**Social stratification**				
**Model 2a**<br>no confounders	77	0.92	0.08	1.74
**Model 2b**<br>+ phylo	77	0.82	-0.10	1.75
**Model 2c**<br>+ space [rho = 0.02]	77	0.89	-0.11	1.88
**Model 2d**<br>+ phylo + space [rho = 0.02]	77	0.84	-0.19	1.87
**Model 2e**<br>+ atolls	71	1.20	0.32	2.11
**Model 2f**<br>+ atolls + phylo + space [rho = 0.02]	71	1.01	-0.15	2.12
**Model 2g**<br>+ space [rho = 0.04]	77	0.71	-0.41	1.73
**Model 2h**<br>+ space [rho = 0.06]	77	0.60	-0.55	1.66
**Model 2i**<br>+ space [rho = 0.08]	77	0.52	-0.63	1.59

Table S2. Differences in political complexity and social stratification levels based on model predictions in the presence versus absence of kava. All models predict an average increase in political complexity and social stratification in the presence of kava, ranging from 0.34 to 0.83 units on the original 5-point scale. However, the 95% credible intervals are relatively wide. Models correspond to Figure 2 and Table S1.

Model	Median	2.5% CI	97.5% CI
**Political complexity**			
Model 1a	0.73	0.29	1.17
Model 1b	0.43	-0.02	0.89
Model 1c	0.45	0.01	0.91
Model 1d	0.42	0.00	0.87
Model 1e	0.83	0.36	1.31
Model 1f	0.45	-0.07	0.97
**Social stratification**			
Model 2a	0.44	0.04	0.83
Model 2b	0.37	-0.04	0.80
Model 2c	0.37	-0.05	0.83
Model 2d	0.34	-0.08	0.78
Model 2e	0.56	0.15	0.98
Model 2f	0.41	-0.06	0.90

Table S3. The posterior median, 95% credible intervals and posterior probabilities (PP) for Δθ, that is the change in the equilibrium trait value for one variable which results from a median absolute deviation increase in another variable. The results of models with posterior tree, eta_anc prior = normal(-2, 1) and different spatial covariance parameters (rho).

**Model**	**Effect**	**Median**	**2.5% CI**	**97.5% CI**	**PP**
**Political complexity (PolC)**					
**Model 0**<br>no distance matrix	Kava → PolC	1.19	-3.10	9.30	0.88
	PolC → Kava	0.84	-8.77	8.80	0.80
**Model 1**<br>rho = normal(0.01, 0.01)	Kava → PolC	0.02	-8.62	3.48	0.51
	PolC → Kava	0.22	-6.31	4.02	0.59
**Model 2**<br>rho = normal(0.02, 0.02)	Kava → PolC	-0.14	-10.24	3.13	0.45
	PolC → Kava	0.19	-7.25	4.58	0.57
**Social stratification (SocS)**					
**Model 0**<br>no distance matrix	Kava → SocS	0.49	-12.64	4.69	0.65
	SocS → Kava	0.67	-6.68	6.99	0.77
**Model 1**<br>rho = normal(0.01, 0.01)	Kava → SocS	-0.11	-10.08	3.16	0.47
	SocS → Kava	0.28	-6.41	4.22	0.60
**Model 2**<br>rho = normal(0.02, 0.02)	Kava → SocS	-0.16	-9.33	3.46	0.45
	SocS → Kava	0.13	-7.84	4.03	0.55

Table S4. The posterior median, 95% credible intervals and posterior probabilities (PP) for Δθ, that is the change in the equilibrium trait value for one variable which results from a median absolute deviation increase in another variable. The results of models with posterior tree, default eta_anc prior = std_normal() and different spatial covariance parameters (rho).

Model	Effect	Median	2.5% CI	97.5% CI	PP
**Political complexity (PolC)**					
**Model 0**<br>no distance matrix	Kava → PolC	1.74	-2.07	20.29	0.92
	PolC → Kava	2.01	-1.07	36.60	0.95
**Model 1**<br>rho = normal(0.01, 0.01)	Kava → PolC	0.30	-11.45	10.02	0.57
	PolC → Kava	0.37	-10.62	10.65	0.59
**Model 2**<br>rho = normal(0.02, 0.02)	Kava → PolC	0.11	-14.67	11.37	0.53
	PolC → Kava	0.22	-15.48	9.02	0.55
**Social stratification (SocS)**					
**Model 0**<br>no distance matrix	Kava → SocS	1.04	-8.58	12.25	0.80
	SocS → Kava	1.38	-9.47	30.86	0.83
**Model 1**<br>rho = normal(0.01, 0.01)	Kava → SocS	0.36	-11.28	8.87	0.59
	SocS → Kava	0.32	-12.74	13.65	0.58
**Model 2**<br>rho = normal(0.02, 0.02)	Kava → SocS	0.16	-12.01	10.79	0.54
	SocS → Kava	0.16	-10.70	9.65	0.54

Table S5. The posterior median, 95% credible intervals and posterior probabilities (PP) for Δθ, that is the change in the equilibrium trait value for one variable which results from a median absolute deviation increase in another variable. The results of models with summary tree, eta_anc prior = normal(-2, 1) and different spatial covariance parameters (rho).

Model	Effect	Median	2.5% CI	97.5% CI	PP
**Political complexity**					
**Model 0** no distance matrix	Kava → PolC	1.99	-0.95	32.51	0.96
	PolC → Kava	1.59	-5.09	25.11	0.88
**Model 1** rho = normal(0.01, 0.01)	Kava → PolC	1.99	-0.73	28.50	0.96
	PolC → Kava	1.74	-3.68	33.64	0.90
**Model 2** rho = normal(0.02, 0.02)	Kava → PolC	1.39	-7.89	24.06	0.79
	PolC → Kava	1.11	-7.78	23.28	0.75
**Social stratification**					
**Model 0** no distance matrix	Kava → SocS	1.29	-9.66	13.08	0.83
	SocS → Kava	1.46	-7.45	33.64	0.84
**Model 1** rho = normal(0.01, 0.01)	Kava → SocS	1.16	-11.54	18.16	0.78
	SocS → Kava	1.18	-6.13	29.05	0.81
**Model 2** rho = normal(0.02, 0.02)	Kava → SocS	0.52	-14.57	10.80	0.61
	SocS → Kava	0.64	-16.98	19.57	0.66

Table S6. The posterior median, 95% credible intervals and posterior probabilities (PP) for Δθ, that is the change in the equilibrium trait value for one variable which results from a median absolute deviation increase in another variable. The results of models with summary tree, default eta_anc prior = std_normal() and different spatial covariance parameters (rho).

Model	Effect	Median	2.5% CI	97.5% CI	PP
**Political complexity (PolC)**					
**Model 0**<br>no distance matrix	Kava → PolC	2.80	0.21	66.46	0.98
	PolC → Kava	2.68	-1.48	44.37	0.95
**Model 1**<br>rho = normal(0.01, 0.01)	Kava → PolC	2.93	0.06	50.23	0.98
	PolC → Kava	2.98	-1.55	53.05	0.96
**Model 2**<br>rho = normal(0.02, 0.02)	Kava → PolC	1.95	-9.36	32.42	0.82
	PolC → Kava	1.92	-6.59	58.50	0.83
**Social stratification (SocS)**					
**Model 0**<br>no distance matrix	Kava → SocS	1.73	-7.08	20.82	0.88
	SocS → Kava	2.46	-5.42	52.04	0.92
**Model 1**<br>rho = normal(0.01, 0.01)	Kava → SocS	1.69	-9.93	25.42	0.84
	SocS → Kava	2.04	-7.57	42.94	0.87
**Model 2**<br>rho = normal(0.02, 0.02)	Kava → SocS	1.09	-17.74	23.33	0.69
	SocS → Kava	1.19	-13.23	41.58	0.69

Table S7. Summary cross-table of traditional kava drinking in relation to atolls (N = 83).

	Atolls		
Kava drinking	No	Almost-atoll	Yes
Absent	35	6	11
Present	25	1	0
NA	*4*	*0*	*1*

Table S8. Summary cross-table of traditional kava drinking in relation to levels of political complexity (N = 83).

	Political complexity				
Kava drinking	0	1	2	3	4
Absent	14	21	15	2	0
Present	4	3	11	6	2
NA	*1*	*2*	*2*	*0*	*0*

Table S9. Summary cross-table of traditional kava drinking in relation to levels of social stratification (N = 83).

	Social stratification					
Kava drinking	0	1	2	3	4	*NA*
Absent	4	27	18	0	2	*1*
Present	1	8	12	1	4	*0*
NA	*0*	*2*	*3*	*0*	*0*	*0*

Readme for Dataset S1

The following includes descriptions for each column in the Dataset S1.
Each row represents one society.

COLUMNS

Language:	Language name according to Gray et al.'s (1) phylogeny.
Glottocode:	Glottocode of language.
Latitude:	Latitude of society.
Longitude:	Longitude of society.
ABVD:	Language ID in Austronesian Basic Vocabulary Database.
Isocode:	Language ISO code.
Atoll:	Does society live on atoll(s): 0 = No, 1 = Yes, 0.5 = uplifted atolls and almost-atolls.
Society:	The name of the society or island/region.
Kava_Drinking:	Kava drinking at the time of European contact. 0 = Absent; 1 = Present; NA = Insufficient information or conflicting information.
Kava_References:	The references from which information on the kava is obtained.
Kava_Notes:	Summary of information on the kava.
Political_Complexity:	The number of jurisdictional levels beyond the local community.<br>0 = Politically uncentralized even at the community level;<br>1 = Politically centralized at the community level only (e.g., autonomous bands and villages);<br>2 = One level above the local community (e.g., simple chiefdoms);<br>3 = Two levels above the local community (e.g., complex chiefdoms);<br>4 = Three or more levels above the local community (e.g., states)
Social_Stratification:	Social stratification according to Murdock & Provost's (3) scale.<br>0 = Egalitarian;<br>1 = Wealth distinctions and/or hereditary slavery;<br>2 = Two social classes, but no caste divisions or hereditary slavery,<br>3 = Two social classes, with caste divisions and/or hereditary slavery;<br>4 = Three or more social classes or castes, with or without hereditary slavery.
Focal_Time:	Approximate year to which data on kava drinking and sociopolitical complexity refer.
Focal_Time_Refer.	The references from which information on the focal time is obtained.
Focal_Time_Notes:	Notes regarding focal time.
PC_SS_References:	The references from which information on the political complexity and social stratification is obtained.
PC_SS_Notes:	Summary of information on political complexity and social stratification, and justification of coding decisions.
Region:	The region of society (loosely defined).
Cultural_Area:	The classification of society in the three major Pacific cultural areas (Melanesia, Micronesia, Polynesia), including the category of Polynesian outliers.

Full References for Dataset S1

Adams, R. (1984). *In the land of strangers: A century of European contact with Tanna, 1774-1874*. Australian National University Press.

Aitken, R. T. (1930). *Ethnology of Tubuai*. Bernice P. Bishop Museum.

Albert, S. M. (1991). Lak. In T. E. Hays (Ed.), *Encyclopedia of World Cultures* (Vol. II, pp. 137-139). G.K. Hall & Co.

Aswani, S. (2008). Forms of Leadership and violence in Malaita and in the New Georgia group, Solomon Islands. In P. J. Stewart, & A. Strathern (Eds.), *Exchange and sacrifice*. Carolina Academic Press.

Athens, J. S. (2007). Prehistoric population growth on Kosrae, eastern Caroline Islands. In P. V. Kirch & J. Rallu, *The Growth and Collapse of Pacific Island Societies*. University of Hawaii Press.

Ballara, A. (1998). *Iwi: The dynamics of Māori tribal organisation from c.1769 to c.1945*. Victoria University Press.

Baltaxe, J. B. (1975). *The transformation of the rangatira: A case of the European reinterpretation of Rarotongan social organization* (Unpublished doctoral dissertation, University of Illinois).

Bedford, R., Macdonald, B., & Munro, D. (1980). Population estimates for Kiribati and Tuvalu, 1850-1900: Review and speculation. *The Journal of the Polynesian Society*, **89**(2), 199-246.

Bennett, J. A. (1974). *Cross-Cultural influences on village relocation on the Weather Coast of Guadalcanal, Solomon Islands, c.1870-1953* (Masters Thesis, University of Hawai'i). ScholarSpace. https://scholarspace.manoa.hawaii.edu/items/23cd2b3d-4fe0-4d3f-a247-508797b031c3.

Birket-Smith, K. (1956). *An ethnological sketch of Rennell Island*. Det Kongelige Danske Videnskabernees Selskab.

Blackwood, B. (1935). *Both Sides of Buka Passage: An Ethnographic Study of Social, Sexual and Economic Questions in the North-western Solomon Islands*. Clarendon Press.

Blythe, J. M. (1978). *Following both sides: Processes of group formation in Vitu* (Unpublished doctoral disseration). McMaster University.

Boltt, R. (2008). Excavations in Peva Valley, Rurutu, Austral Islands (East Polynesia). *Asian Perspectives*, **47**(1), 158-187. https://www.jstor.org/stable/42928737.

Brunton, R. (1989). *The abandoned narcotic: kava and cultural instability in Melanesia*. Cambridge University Press.

Buck, P. H. (1932). *Ethnology of Tongareva*. Bernice P. Bishop Museum. https://ndhadeliver.natlib.govt.nz/webarchive/20210104000423/http://nzetc.victoria.ac.nz/tm/scholarly/tei-BucTong.html.

Buck, P. H. (1938). *Ethnology of Mangareva*. Bernice P. Bishop Museum.

Buck, P. H. (1949). *The coming of the Maori*. Māori Purposes Fund Board. https://ndhadeliver.natlib.govt.nz/webarchive/20210104000423/http://nzetc.victoria.ac.nz/tm/scholarly/tei-BucTheC.html.

Burrows, E. G. (1936). *Ethnology of Futuna*. Bernice P. Bishop Museum.

Burrows, E. G. (1937). *Ethnology of Uvea (Wallis Island)*. Bernice P. Bishop Museum.

Burt, B. (1994). *Tradition and Christianity: The Colonial Transformation of a Solomon Islands Society*. Harwood Academic Publishers.

Capell, A. (1958). *The Culture and Language of Futuna and Aniwa, New Hebrides*. University of Sydney.

Carroll, V. (1966). *Nukuoro kinship* (Unpublished doctoral dissertation, University of Chicago).

Carroll, V. (1975). The population of Nukuoro in historical perspective. In V. Carroll (Ed.), *Pacific Atoll Populations*. University of Hawaii Press.

Chowning, A. (1980). Culture and biology among the Sengseng of New Britain. *The Journal of the Polynesian Society*, **89**(1), 7-31. https://www.jstor.org/stable/20705459.

Chowning, A. (1991a). Lakalai. In T. E. Hays (Ed.), *Encyclopedia of World Cultures* (Vol. II). G.K. Hall & Co.

Chowning, A. (1991b). Sengseng. In T. E. Hays (Ed.), *Encyclopedia of World Cultures* (Vol. II). G.K. Hall & Co.

Chowning, A. & Goodenough, W. H. (1971). Lakalai political organization. In R. M. Berndt & P. Lawrence (Eds.), *Politics in New Guinea*. University of Western Australia Press.
Codrington, R.H. (1891). *The Melanesians: Studies in their anthropology and folklore*. Clarendon Press. https://archive.org/details/melanesiansstudi00codruoft.
Davenport, W. H. (1964). Social Structure of Santa Cruz. In W. Goodenough (Ed.), *Explorations in Cultural Anthropology: Essays in Honour of George Peter Murdock*. McGraw-Hill.
Davenport, W. H. (1968). Social organization notes on the northern Santa Cruz Islands: the Duff Islands (Taumako). *Baessler-Archiv, Neue Folge* 16, 137-205.
Davenport, W. H. (1969a). Social organization notes on the Southern Santa Cruz Islands: Utupua and Vanikoro. *Baessler-Archiv; Beiträge Zur Völkerkunde*, **16**(41), 207-275.
Davenport, W. H. (1969b). Social organization notes on the Northern Santa Cruz Islands: the Main Reef Islands. *Baessler-Archiv, Neue Folge* **17**(1): 151–243.
Deacon, B. (1934). *Malekula: A Vanishing People in the New Hebrides*. George Routledge and Sons.
Doumenge, J. (1974). *Paysans Melanésiens en Pays Canala Nouvelle Calédonie*. Centre d'Études de Géographie Tropicale, Domaine Universitaire de Bordeaux.
Dubois, M. (1984). *Gens de Maré*. Éditions Anthropos.
Edwards, E. (2003). *Archaeological survey of Ra'ivavae*. Bearsville Press for the Easter Island Foundation.
Emory, K. P. (1965). *Kapingamarangi: Social and religious life of a Polynesian atoll*. Bernice P. Bishop Museum.
Emory, K. P. (1975). *Material culture of the Tuamotu Archipelago*. Bernice P. Bishop Museum.
Epstein, T. S. (1968). *Capitalism, Primitive and Modern: Some Aspects of Tolai Economic Growth*. Australian National University Press.
Erdland, A. (1961). *The Marshall Islanders: Life and customs, thought and religion of a South Seas people*. (R. Neuse, Trans.). New Haven, CT: Human Relations Area Files. (Originally work published 1914).
Facey, E. E. (1982). *Ideology and identity: Social construction of reality on Nguna, Vanuatu* (Doctoral dissertation, University of Sydney). Sydney Digital Theses. http://hdl.handle.net/2123/8600.
Feinberg, R. (2004). *Anuta: Polynesian lifeways for the 21st century*. Waveland Press.
Ferdon, E. N. (1987). *Early Tonga*. The University of Arizona Press.
Firth, R. (1936). *We, the Tikopia: A sociological study of kinship in primitive Polynesia*. Stanford University Press.
Firth, R. (1959). *Social Change in Tikopia: Re-Study of a Polynesian Community after a Generation*. Allen and Unwin.
Fison, L. (1881). Land tenure in Fiji. *The Journal of the Anthropological Institute of Great Britain and Ireland*, **10**, 332-352. https://www.jstor.org/stable/2841531.
Gardiner, J. S. (1898). The natives of Rotuma. *The Journal of the Anthropological Institute of Great Britain and Ireland*, **27**, 396-435. https://www.jstor.org/stable/2842839.
Goldie, Rev, J.F. (1909). The People of New Georgia. Their Manners and Customs, and Religious Beliefs. *Proceedings of the Royal Society of Queensland*, **22**, 23-30. Retrieved from: https://archive.org/details/proceedingsofroy2225roya/page/n30/mode/1up.
Goodenough, W. H. (2002). *Under heaven's brow: Pre-Christian religious tradition in Chuuk*. American Philosophical Society.
Graves, M. W. (1986). Late Prehistoric Complexity on Lelū: Alternatives to Cordy's Model. *The Journal of the Polynesian Society*, **95**(4), 479-489. https://www.jstor.org/stable/20706034.
Grimble, A. F. (1989). *Tungaru traditions: Writings on the atoll aulture of the Gilbert Islands*. University of Hawaii Press.
Groves, W. C. (1934). Tabar to-day: A study of a Melanesian community in contact with alien non-primitive cultural forces. *Oceania*, **5**(2), 224-240. https://www.jstor.org/stable/40327832.
Groves, M. (1963). Western Motu descent groups. *Ethnology*, **2**(1), 15-30. https://www.jstor.org/stable/3772965.

Gunn, W. (1914). *The Gospel in Futuna*. Hodder & Stoughton. https://dn790001.ca.archive.org/0/items/gospelinfutunawi00gunniala/gospelinfutunawi00gunniala.pdf.
Handy, E.S.C. (1923). *The native culture in the Marquesas*. Bernice P. Bishop Museum. https://archive.org/details/nativecultureinm00hand.
Hanlon, D. (1988). *Upon a Stone Altar: A History of the Island of Pohnpei to 1890*. University of Hawaii Press.
Hannemann, E. F. (1996). *Village Life and Social Change in Yam Society*. Kristen Pres.
Hanson, F. A. (1970). *Rapan lifeways; society and history on a Polynesian island*. Little, Brown & Company.
Hogbin, H. I. (1934). *Law and order in Polynesia: A study of primitive legal institutions*. Christophers.
Hogbin, H. I. (1939a). *Experiments in Civilization: The Effects of European Culture on a Native Community of the Solomon Islands*. George Routledge and Sons.
Hogbin, H. I. (1978). *The Leaders and the Led: Social Control in Wogeo, New Guinea*. Melbourne University Press.
Howard, A. (1964). Land tenure and social change in Rotuma. *The Journal of the Polynesian Society*, **73**(1), 26-52. https://www.jstor.org/stable/20704149.
Humphreys, C. B. (1926). *The Southern New Hebrides: An Ethnological Record*. Cambridge, UK: Cambridge University Press. https://archive.org/details/southernnewhebri0000hump.
Hviding, E. (1996). *Guardians of Marovo Lagoon: Practice, Place, and Politics in Maritime Melanesia*. University of Hawaii Press.
Ivens, W. G. (1927*). Melanesians of the South-East Solomon Islands*. Benjamin Blom.
Ivens, W. G. (1930). *The Island Builders of the Pacific*. Seeley, Service & Co. Ltd.
Jenness, D. & Ballantyne, A. (1920). *The Northern D'Entrecasteaux*. Clarendon Press.
Keesing, R. M. (1982). *Kwaio religion: The living and the dead in a Solomon Island society*. Columbia University Press.
Keesing, R. M. (1985). Killers, big men, and priests on Malaita: Reflections on a Melanesian troika system. *Ethnology*, **24**, 237-252. https://www.jstor.org/stable/3773736.
King, M. (2003). *The Penguin history of New Zealand*. Penguin Group (NZ).
Kirby, K. R., Gray, R. D., Greenhill, S. J., Jordan, F. M., Gomes-Ng, S., Bibiko, H. J., ... & Leehr, D. (2016). D-PLACE: A global database of cultural, linguistic and environmental diversity. *PLoS One*, **11**(7), e0158391. https://doi.org/10.1371/journal.pone.0158391
Kirch, P. V. (2010). *How chiefs became kings: Divine kingship and the rise of archaic states in ancient Hawai'i*. University of California Press.
Lebot, V., Merlin, M. & Lindstrom, L. (1997). *Kava: The Pacific Elixir*. Healing Arts Press.
Lutkehaus, N. C. (1995). *Zaria’s Fire: Engendered moments in Manam ethnography*. Carolina Academic Press.
Macdonald, B. (1982). *Cinderellas of the Empire: Towards a history of Kiribati and Tuvalu*. Australian National University Press.
MacGregor, G. (1937). *Ethnology of Tokelau Islands*. Bernice P. Bishop Museum. https://ndhadeliver.natlib.govt.nz/webarchive/20210104000423/http://nzetc.victoria.ac.nz/tm/scholarly/tei-MacToke.html.
Malinowsi, B. (1922). *Argonauts of the Western Pacific: An account of native enterprise and adventure in the archipelagoes of Melanesian New Guinea*. Routledge & Sons. https://archive.org/details/in.gov.ignca.15655.
Mason, L. E. (1947). *The economic organization of the Marshall Islanders*. U.S. Commercial Company.
McArthur, N. (1967). *Island populations of the Pacific*. Australian National University Press.
Métraux, A. (1940). *Ethnology of Easter Island*. Bernice P. Bishop Museum Press.
Nachman, S. (1982). The validation of leadership on Nissan. *Oceania*, **52**(3), 199-220. https://www.jstor.org/stable/40330611.
Nevermann, H. (2010). *St. Matthias Group*. Dunedin, New Zealand: University of Otago (J. Dennison, Trans, Original work published 1933).
Oliver, D.L. (1974). *Ancient Tahitian society*. The University Press of Hawaii.

Pitt-Rivers, G. L. F. (1925). Aua island: Ethnological and sociological features of a South Sea pagan society. *The Journal of the Royal Anthropological Institute of Great Britain and Ireland*, **55**, 425-438. https://www.jstor.org/stable/2843649.

Ray, S. H. (1917). The people and language of Lifu, Loyalty Islands. *The Journal of the Royal Anthropological Institute of Great Britain and Ireland*, **47**, 239-322. https://www.jstor.org/stable/2843343.

Rivers, W. H. R. (1914). *The history of Melanesian society. Volume I*. Cambridge University Press. https://archive.org/details/b31362692_0001/mode/2up.

Roscoe, P. B. (1991). Tongareva. In T. E. Hays (Ed.), *Encyclopedia of World Cultures* (Vol. II). G.K. Hall & Co.

Routledge, D. (1991). Bau. In T. E. Hays (Ed.), *Encyclopedia of World Cultures* (Vol. II). G.K. Hall & Co.

Ryan, T. F. (1977). *Prehistoric Niue: An egalitarian Polynesian society* (Unpublished Masters Thesis, University of Auckland).

Scheffler, H. W. (1962). Kindred and kin groups in Simbo Island social structure. *Ethnology*, **1**(2), 135-157. https://www.jstor.org/stable/3772871.

Scheffler, H. W. (1965). *Choiseul Island social structure*. University of California Press.

Schwartz, T. (1963). Systems of areal integration: Some considerations based on the Admiralty Islands of Northern Melanesia. *Anthropological Forum*, **1**(1), 56-97. https://doi.org/10.1080/00664677.1963.9967181

Seligman, C. G. (1910). *The Melanesians of British New Guinea*. Cambridge University Press. https://archive.org/details/melanesiansofbri00seli.

Senfft, A. (1942). The Marshall Islanders (J. Springer, Trans.). eHRAF World Cultures. *Human Relations Area Files*. https://ehrafworldcultures.yale.edu/cultures/or11/documents/011 (Original work published 1903 as "Die Marshall-Insulaner" in S. R. Steinmetz (Ed.), *Rechtsverhältnisse von eingeborenen Völkern in Afrika und Ozeanien* (pp. 425–455). J. Springer.)

Smith, M.F. (1994). *Hard times on Kairiru Island*. University of Hawaii Press.

Spriggs, M. 1981). *Vegetable kingdoms: Taro irrigation and Pacific prehistory* (Unpublished doctoral dissertation, Australian National University).

Spriggs, M., & Wickler, S. (1989). Archaeological research on Erromango: Recent data on Southern Melanesian prehistory. *Bulletin of the Indo-Pacific Prehistory Association*, **9**, 68-91.

Stair, J. B. (1897). *Old Samoa, or, flotsam and jetsam from the Pacific Ocean*. The Religious Tract Society. https://archive.org/details/oldsamoaorflots00staigoog/mode/2up.

Stephen, M. (1974). *Continuity and change in Mekeo society, 1890-1971* (Doctoral dissertation, Australian National University). Open Research Repository. https://openresearch-repository.anu.edu.au/items/007bdacb-19f9-4cb4-b583-1f2dc849fff4.

Tolerton, B., & Rauch, J. (1949). *Social organization, land tenure and subsistence economy of Lukunor, Nomoi Islands* (CIMA Report No. 26). Office of Naval Research; National Academy of Sciences.

Tonkinson, R. (1968). *Maat Village, Efate: A relocated community in the New Hebrides*. University of Oregon.

Watts, J., Sheehan, O., Greenhill, S. J., Gomes-Ng, S., Atkinson, Q. D., Bulbulia, J., & Gray, R. D. (2015b). Pulotu: database of Austronesian supernatural beliefs and practices. *PLoS One*, 10(9), e0136783. https://doi.org/10.1371/journal.pone.0136783.

Weiner, A. B. (1988). *The Trobrianders of Papua New Guinea*. Holt, Rinehart and Winston, Inc.

White, G. M. (1991). *Identity through history: Living stories in a Solomon Islands society*. Cambridge University Press.

Young, M. W. (1991a). Dobu. In T. E. Hays (Ed.), *Encyclopedia of World Cultures* (Vol. II). G.K. Hall & Co.

Young, M. W. (1991b). Goodenough Island. In T. E. Hays (Ed.), *Encyclopedia of World Cultures* (Vol. II). G.K. Hall & Co.

Zelenietz, M. (1991). Kilenge. In T. E. Hays (Ed.), *Encyclopedia of World Cultures* (Vol. II). G.K. Hall & Co.

SI References

1. R. D. Gray, A. J. Drummond, S. J. Greenhill, Language phylogenies reveal expansion pulses and pauses in Pacific settlement. *Science* **323**, 479-483 (2009).
2. H. Skirgård *et al.*, Grambank reveals the importance of genealogical constraints on linguistic diversity and highlights the impact of language loss. *Science Advances* **9**, eadg6175 (2023).
3. G. P. Murdock, C. Provost, Measurement of Cultural Complexity. *Ethnology* **12**, 379-392 (1973).

Other supporting materials

Dataset S1 (separate file). A dataset on kava drinking, political complexity, social stratification and location on atolls.
